# Supplementary material for: The Antigenic Topology of Norovirus as Defined by B and T Cell Epitope Mapping: Implications for Universal Vaccines and Therapeutics
Source: Viruses. 2019 May 10;11(5):432. doi: 10.3390/v11050432 (PMC6563215; doi:10.3390/v11050432)
Supplement: Supplementary file 1 [file viruses-11-00432-s001.pdf]

Table S1. mAbs that react to GI VLPs.

Antibody	Isotype	Source	Initial mode of immunization	Immunogen genotype (Strain)	Immunogen (Origin)	Recognizes (Strain)	Reactive region (Epitope)	Epitope arrangement	Amino acid region	Ref(s)
1C9	IgG1	Mouse	IP injection	GI.1	Live virus from stool filtrate	GI.1	S domain	Linear	-	[239]
1D8	IgG1	Mouse	IP injection	GI.1	Live virus from stool filtrate	GI.1	S domain	Linear	-	[239]
NV834	IgG1	Mouse	IP injection	GI.1	VLP (baculo)	GI.1 (NV [nb], SEV)	S domain	Conformational	(*Identical to NV813*)	[92, 240-243]
NV142	IgG1	Mouse	IP injection	GI.1	VLP (baculo)	GI.1 [nb]	S domain	Conformational	-	[92, 240, 241, 243]
NV101	IgG1	Mouse	IP injection	GI.1	VLP (baculo)	GI.1 [nb]	P domain	Conformational	(*Similar to NV813 and NV834*)	[92, 240, 241, 243]
NV813	IgG1	Mouse	IP injection	GI.1	VLP (baculo)	GI.1 [nb]	P domain	Conformational	(*Identical to NV834*)	[92, 240, 241, 243]
NV7411	IgG1	Mouse	IP injection	GI.1	VLP (baculo)	GI.1 [nb]	P domain	Conformational	(*Similar to NV3901, NV3912, NV2461*)	[92, 240, 241, 243]
NV2461	IgG1	Mouse	IP injection	GI.1	VLP (baculo)	GI.1 (NV [nb], SEV), GI.2, GI.4	P1 domain	Linear	Between 457-530	[92, 240-243]
NV8301	IgG1	Mouse	IP injection	GI.1	VLP (baculo)	GI.1 [H]	P domain	Conformational	(*Similar to NV8812*)	[92, 240, 241, 243]
4E6	IgG (k)	Mouse	Subcut injection	GI.1	VP1 (bacteria)	GI.1, GI.4	S domain	Linear	Between 1-43 [1-30]	[244]
4F7	IgG (k)	Mouse	Subcut injection	GI.1	VP1 (bacteria)	GI.1	S domain	Linear	Between 1-43	[244]
7D2	IgG (k)	Mouse	Subcut injection	GI.1	VP1 (bacteria)	GI.1	S domain	Linear	Between 1-43	[244]
7E9	IgG (k)	Mouse	Subcut injection	GI.1	VP1 (bacteria)	GI.1	S domain	Conformational	-	[244]
13F9	IgG (k)	Mouse	Subcut injection	GI.1	VP1 (bacteria)	GI.1	S domain	Linear	Between 1-43	[244]
1G9	-	Mouse	Subcut injection	GI.1	VP1 (bacteria)	GI.1	S domain	Linear	Between 1-43	[244]
3D10	-	Mouse	Subcut injection	GI.1	VP1 (bacteria)	GI.1	S domain	Linear	Between 1-43	[244]
4B10	-	Mouse	Subcut injection	GI.1	VP1 (bacteria)	GI.1	S domain	Linear	Between 1-43	[244]
5B6	-	Mouse	Subcut injection	GI.1	VP1 (bacteria)	GI.1	P2 domain	Linear	Between 292-342 [318-326: *GFPDLG*C]	[244]
6C4	-	Mouse	Subcut injection	GI.1	VP1 (bacteria)	GI.1	S domain	Linear	Between 1-43	[244]
8C7	-	Mouse	Subcut injection	GI.1	VP1 (bacteria)	GI.1, GI.4	P1 domain	Linear	Between 451-489	[244]
8D2	-	Mouse	Subcut injection	GI.1	VP1 (bacteria)	GI.1	S domain	Linear	Between 1-43	[244]
10F8	-	Mouse	Subcut injection	GI.1	VP1 (bacteria)	GI.1	S domain	Linear	Between 1-43	[244]
10G10	-	Mouse	Subcut injection	GI.1	VP1 (bacteria)	GI.1	P2 domain	Linear	Between 292-342	[244]
12D4	-	Mouse	Subcut injection	GI.1	VP1 (bacteria)	GI.1	S domain	Linear	Between 1-43	[244]
15G5	-	Mouse	Subcut injection	GI.1	VP1 (bacteria)	GI.1	S domain	Linear	Between 1-43	[244]
NV51	IgA	Mouse	Oral	GI.1	VLP (baculo)	GI.1 (NV, SEV), GI.2, GI.4	P domain	Linear	-	[242]
NV138	IgA	Mouse	Oral	GI.1	VLP (baculo)	GI.1 (NV, SEV), GI.4	P domain	Linear	-	[242]
NV172	IgA	Mouse	Oral	GI.1	VLP (baculo)	GI.1 (NV, SEV), GI.4	-	Linear	-	[242]
NV5610	IgA	Mouse	Oral	GI.1	VLP (baculo)	GI.1 (NV, SEV)	-	Conformational	-	[242]
NV5620	IgA	Mouse	Oral	GI.1	VLP (baculo)	GI.1 (NV, SEV)	-	Conformational	-	[242]
mAb 54.6	IgG, scFv	Mouse	IP injection	GI.1	VLP (baculo)	GI.1 [B,H]	P2 domain	Conformational	T280, R291, G292, T293, T302	[163, 164]
107-5	IgG	Mouse	IP injection	GI.4 + GII.6	VLP (baculo)	GI.1, GI.3, GI.4, GI.7, GI.8, GI.12	-	-	-	[195]
B7	IgG, Fab	Chimp	Oral	GI.1	Live virus	GI.1 (NV [B,H,N], SW-2007), GI.6	P domain – Epitope group 1	Conformational (hetero-oligomeric)	-	[88]
D8	IgG, Fab	Chimp	Oral	GI.1	Live virus	GI.1 (NV [B,H,N], SW-2007)	P domain – Epitope group 2	Conformational (hetero-oligomeric)	-	[88]
E5	IgG, Fab	Chimp	Oral	GI.1	Live virus	GI.1 (NV [B,H], SW-2007)	P domain – Epitope group 2	Conformational (hetero-oligomeric)	-	[88]
G4	IgG, Fab	Chimp	Oral	GI.1	Live virus	GI.1 (NV [B,H])	P domain – Epitope group 3	Conformational (hetero-oligomeric)	Includes G365	[88]
F11	IgG, Fab	Chimp	Oral	GI.1	Live virus	GI.1 (NV [B,H], SW-2007)	P domain – Epitope group 4	Conformational	-	[88]

CV-1A1	scFv	Human	(phage display selection)	GI.4	VLP (baculo)	GI.4 ^{B}	-	-	-	[245]
CV-2F5	scFv	Human	(phage display selection)	GI.4	VLP (baculo)	GI.1, GI.2, GI.3, GI.4 ^{B}, GI.6	-	-	-	[245]
1A8	IgG	Human	Oral – artificial challenge (PBMC-generated)	GI.1	Live virus	GI.1 ^{B}	P domain	Conformational	-	[124, 246]
2L8	IgG	Human	Oral – artificial challenge (PBMC-generated)	GI.1	Live virus	GI.1 ^{B}	P domain	Conformational	-	[124, 246]
3I23	IgG (switch to IgA)	Human	Oral – artificial challenge (PBMC-generated)	GI.1	Live virus	GI.1 ^{B,H}	P domain (HBGA binding domain)	Conformational	Includes W375	[124, 246]
4E7	IgG	Human	Oral – artificial challenge (PBMC-generated)	GI.1	Live virus	GI.1 ^{B}	P domain	Conformational	-	[124, 246]
4I23	IgG (switch to IgA)	Human	Oral – artificial challenge (PBMC-generated)	GI.1	Live virus	GI.1 ^{B,H}	P domain (HBGA binding domain)	Conformational	Includes W375	[124, 246]
NV1	IgG	Human	Oral – artificial challenge (PBMC-generated)	GI.1	Live virus	GI.1 ^{B}	-	-	-	[124, 246]
NV48	IgG	Human	Oral – artificial challenge (PBMC-generated)	GI.1	Live virus	GI.1 ^{B}	-	-	-	[124, 246]
2J3	IgA (switch to IgG)	Human	Oral – artificial challenge (PBMC-generated)	GI.1	Live virus	GI.1 ^{B,H}	P domain	Conformational	-	[124, 246]
3I3	IgA	Human	Oral – artificial challenge (PBMC-generated)	GI.1	Live virus	GI.1 ^{B}	P domain	Conformational	-	[124, 246]
4B19	IgA	Human	Oral – artificial challenge (PBMC-generated)	GI.1	Live virus	GI.1 ^{B}	P domain	Conformational	-	[124, 246]
4C10	IgA (switch to IgG)	Human	Oral – artificial challenge (PBMC-generated)	GI.1	Live virus	GI.1 ^{B,H}	P domain	Conformational	-	[124, 246]
5I2	IgA. Fab (switch to IgG)	Human	Oral – artificial challenge (PBMC-generated)	GI.1	Live virus	GI.1 ^{B,H}	P2 domain (loops T, U, Q)	Conformational	D346, T348, D350, T351, F352, S380, H381, S383, N394, G396, S398	[124, 125, 165, 246]
NV41	IgA	Human	Oral – artificial challenge (PBMC-generated)	GI.1	Live virus	GI.1 ^{B}	-	-	-	[124, 246]
NV56	IgA	Human	Oral – artificial challenge (PBMC-generated)	GI.1	Live virus	GI.1 ^{B}	-	-	-	[124, 246]
GI.1A	IgG2a (k)	Mouse	IP injection	GI.1	VLP (VEE)	GI.1 ^{nb}	-	-	-	[121]
GI.1B	IgG2a (k)	Mouse	IP injection	GI.1	VLP (VEE)	GI.1 ^{nb}, GI.3* ^{nb}	-	-	-	[121]
GI.1C	IgM	Mouse	IP injection	GI.1	VLP (VEE)	GI.1 ^{nb}, GI.3* ^{nb}	-	-	-	[121]
GI.1D	IgG2a (k)	Mouse	IP injection	GI.1	VLP (VEE)	GI.1 ^{nb}, GI.3 ^{nb}, GI.4* ^{nb}	-	-	-	[121]
GI.1E	IgG2a (k)	Mouse	IP injection	GI.1	VLP (VEE)	GI.1 ^{nb}	-	-	-	[121]
GI.4A	IgG2a (k)	Mouse	IP injection	GI.4	VLP (VEE)	GI.4 ^{B}	-	-	-	[121]
GI.4B	IgG2a (k)	Mouse	IP injection	GI.4	VLP (VEE)	GI.4 ^{B}	-	-	-	[121]
GI.4C	IgG2a (k)	Mouse	IP injection	GI.4	VLP (VEE)	GI.4 ^{B}	-	-	-	[121]
GI.4D	IgG2a (k)	Mouse	IP injection	GI.4	VLP (VEE)	GI.4 ^{B}	-	-	-	[121]
NVF 144	IgG1	Human	Oral – artificial challenge (PBMC-generated)	GI.1	Live virus	GI.3 ^{B}	-	-	-	[78, 121]
NVB 106	IgG1	Human	Oral – artificial challenge (PBMC-generated)	GI.1	Live virus	GI.1 ^{B}	-	-	-	[78, 121]

NVB 84	IgG1	Human	Oral – artificial challenge (PBMC-generated)	GI.1	Live virus	GI.3 ^{nb}, GI.4 ^{nb}	-	-	-	[78, 121]
NVE 3	IgG1	Human	Oral – artificial challenge (PBMC-generated)	GI.1	Live virus	GI.1 ^{nb}, GI.3* ^{nb}, GI.4* ^{nb}	-	-	-	[78, 121]
N1	VHH	Llama	IM injection	GI.1	VLP (baculo)	GI.1 (NV ^{B,H}, P7-587 ^{H})	P domain	Conformational	-	[102]
N2	VHH	Llama	IM injection	GI.1	VLP (baculo)	GI.1 (NV ^{H}, P7-587 ^{H}), GI.3	P domain	Conformational	-	[102]
N3	VHH	Llama	IM injection	GI.1	VLP (baculo)	GI.1 (NV ^{B}, P7-587 ^{H})	P domain	Conformational	-	[102]
N4	VHH	Llama	IM injection	GI.1	VLP (baculo)	GI.1 (NV ^{B}, P7-587 ^{H})	P domain	Conformational	-	[102]
N5	VHH	Llama	IM injection	GI.1	VLP (baculo)	GI.1 (NV ^{B,H}, P7-587 ^{H})	P domain	Conformational	-	[102]
N6	VHH	Llama	IM injection	GI.1	VLP (baculo)	GI.1 (NV ^{B}, P7-587 ^{nb})	P domain	Conformational	-	[102]
N7	VHH	Llama	IM injection	GI.1	VLP (baculo)	GI.1 (NV ^{B}, P7-587 ^{H})	P domain	Conformational	-	[102]
N8	VHH	Llama	IM injection	GI.1	VLP (baculo)	GI.1 (NV ^{B,H})	P domain	Conformational	-	[102]
N9	VHH	Llama	IM injection	GI.1	VLP (baculo)	GI.1 (NV ^{B,H}, P7-587 ^{H})	P domain	Conformational	-	[102]
N10	VHH	Llama	IM injection	GI.1	VLP (baculo)	GI.1 (NV ^{B,H})	P domain	Conformational	-	[102]
mAb NV1	-	Mouse	Subcut injection	GI.1	VLP (baculo)	GI.1 ^{H}	P domain – Antigenic Site I	Conformational	-	[132]
mAb NV4	-	Mouse	Subcut injection	GI.1	VLP (baculo)	GI.1 ^{H}, GI.3	P domain – Antigenic Site III	Conformational	-	[88, 132]
mAb NV8	-	Mouse	Subcut injection	GI.1	VLP (baculo)	GI.1 ^{H}, GI.3	P domain – Antigenic Site III	Conformational	-	[132]
mAb NV9	-	Mouse	Subcut injection	GI.1	VLP (baculo)	GI.1 ^{H}	P domain – Antigenic Site IV	Conformational	-	[132]
mAb NV10	scFv	Mouse	Subcut injection	GI.1	VLP (baculo)	GI.1 ^{nb}	S domain – Antigenic Site V	Linear	Between 21-32 (A27, S28, <u>D29</u>, P30, L31, A32]	[132]
mAb NV22	-	Mouse	Subcut injection	GI.1	VLP (baculo)	GI.1 ^{nb}	S domain – Antigenic Site V	Linear	21-32: LVPEVNASDPLA	[132]
mAb NV30	-	Mouse	Subcut injection	GI.1	VLP (baculo)	GI.1 ^{H}	P domain – Antigenic Site IV	Conformational	-	[132]
mAb NV40	-	Mouse	Subcut injection	GI.1	VLP (baculo)	GI.1 ^{H}	P domain – Antigenic Site IV	Conformational	-	[132]
mAb NV47	-	Mouse	Subcut injection	GI.1	VLP (baculo)	GI.1 ^{H}	P domain – Antigenic Site II	Conformational	-	[132]
NJT-R3-A1	scFv	Human	(phage display selection)	GI.1	P domain	GI.1*	P domain	-	-	[247]
NJT-R3-A2	scFv	Human	(phage display selection)	GI.1	P domain	GI.1, GI.7*	P domain	-	-	[247]
NJT-R3-A3	scFv	Human	(phage display selection)	GI.1	P domain	GI.1, GI.7*	P domain	-	-	[247]
Nano-7	VHH	Alpaca	Subcut injection	GI.1	VLP (baculo)	GI.1 ^{B}	P domain (dimeric)	Conformational	<u>Chain A:</u> R275, L276, T280, V282, E303, D305, H310, F312, E313, I318, H404, A446, Q449 <u>Chain B:</u> P237, D463, P464, D465, N498, V500	[130]
Nano-62	VHH	Alpaca	Subcut injection	GI.1	VLP (baculo)	GI.1 ^{nb}	P domain (dimeric)	Conformational	<u>Chain A</u>: P237, V462, P464, D465, V500 <u>Chain B:</u> D273, R275, G278, T280, E313, H404, Q449	[130]
Nano-94	VHH	Alpaca	Subcut injection	GI.1	VLP (baculo)	GI.1 ^{B}	P domain (monomeric)	Conformational	Q264, T348, P349, D350, F352, P379, H381, P382, K391, I392, N394, S397, T400, E401	[130]

Table S2. mAbs that react to GII VLPs.

Antibody	Isotype	Source	Initial mode of immunization	Immunogen genotype (Strain)	Immunogen (Origin)	Recognizes (Strain)	Reactive region (Epitope)	Epitope arrangement	Amino acid region	Ref(s)
SM-4	IgM (k)	Mouse	(added to spleen cells)	GII.2	Live virus from stool filtrate	GII.2	(GII.2 SMV VP1)	-	-	[248]
2B3	-	Mouse	Subcut injection	GII.3	VP1 (bacteria)	GII.3, GII.4 (LD*), GII.8	S domain	Linear	Between 181-220	[249]
2F9	-	Mouse	Subcut injection	GII.3	VP1 (bacteria)	GII.3	S domain	Linear?	Between 31-70	[249]
3E6	-	Mouse	Subcut injection	GII.3	VP1 (bacteria)	GII.3	P2 domain	Linear	Between 361-403	[249]
4C3	-	Mouse	Subcut injection	GII.3	VP1 (bacteria)	GII.3	S domain	Linear	Between 31-70	[249]
4D2	IgG1 (k)	Mouse	Subcut injection	GII.3	VP1 (bacteria)	GII.3	P2 domain	Linear	Between 361-403	[249]
4D6	-	Mouse	Subcut injection	GII.3	VP1 (bacteria)	GII.3	S domain	Linear	Between 31-70	[249]
4G4	-	Mouse	Subcut injection	GII.3	VP1 (bacteria)	GII.3	S domain	Linear	Between 31-70	[249]
5C9	-	Mouse	Subcut injection	GII.3	VP1 (bacteria)	GII.3	P2 domain	Linear	Between 361-403	[249]
F44	IgM	Mouse	Oral	GII.13	VLP (baculo)	GI.1*, GII.2*, GII.4 (GV*), GII.13	-	Conformational	-	[242]
F74	IgM	Mouse	Oral	GII.13	VLP (baculo)	GI.1*, GII.2, GII.4 (GV*), GII.13	-	Conformational	-	[242]
NS28	IgG1	Mouse	Oral	GI.1 + GII.2	VLP (baculo)	GI.1*, GII.2, GII.6, GII.4 (NAV, GV), GII.12, GII.13	P domain	Linear	-	[242]
mAb 61.21	IgGa	Mouse	IP injection	GII.2	VLP (baculo)	GII.2 ^{H}	P2 domain	Conformational	T302, 313-22: DIPAPLGVPD	[163, 175]
SwNV3A10	IgG1	Mouse	IP injection	GII.11	VLP (baculo)	GII.4 (Spanish*), GII.11	S domain	Linear	Between 1-216	[250]
SwNV6E11	IgG1	Mouse	IP injection	GII.11	VLP (baculo)	GII.4 (Spanish*), GII.11	S domain	Linear	Between 1-216	[250]
SwNV2C1	IgG1	Mouse	IP injection	GII.11	VLP (baculo)	GII.11	-	Conformational	-	[250]
SwNV6C9	IgG1	Mouse	IP injection	GII.11	VLP (baculo)	GII.11	-	Conformational	-	[250]
SwNV8C12	IgG1	Mouse	IP injection	GII.11	VLP (baculo)	GII.11	-	Conformational	-	[250]
SwNV8E12	IgG1	Mouse	IP injection	GII.11	VLP (baculo)	GII.11	-	Conformational	-	[250]
SwNV8F10	IgG1	Mouse	IP injection	GII.11	VLP (baculo)	GII.11	-	Conformational	-	[250]
SwNV8B10	IgG1	Mouse	IP injection	GII.11	VLP (baculo)	GII.11	-	Conformational	-	[250]
N4B1	IgG2a	Mouse	IP injection	GII.4 (NVgz01)	VP1 (bacteria)	GII.4 (NVgz01)	S domain	Linear	W55, I56, R57, N58, N59, F60	[251]
N7C2	IgM	Mouse	IP injection	GII.4 (NVgz01)	VP1 (bacteria)	GII.4 (NVgz01)	-	Conformational	-	[251]
N8A9	IgM	Mouse	IP injection	GII.4 (NVgz01)	VP1 (bacteria)	GII.4 (NVgz01)	-	-	-	[251]
GII4v0.5	IgG1	Mouse	IP injection	GII.4 (v0_2000)	VLP (baculo)	GII.4 (v0_2000)	P domain ["Sites A + B"]	Conformational	S296, H297, D298, D393, H394, Q395	[34]
GII4v0.8 (αv0.8)	IgG2a	Mouse	IP injection	GII.4 (v0_2000)	VLP (baculo)	GII.4 (VA387 1996, V0-1999*, v0_2000, 2004 ^{B})	P domain ["Sites A + B"]	Conformational	S296, H297, D298, D393, H394, Q395 (*influenced by 397, 448*)	[34, 149, 169]
GII4v0.10	IgG2b	Mouse	IP injection	GII.4 (v0_2000)	VLP (baculo)	GII.4 (v0_2000)	P domain ["Sites A + B"]	Conformational	S296, H297, D298, D393, H394, Q395	[34]
GII4v2.5 (αv2.5)	IgG1	Mouse	IP injection	GII.4 (v2_2004)	VLP (baculo)	GII.4 (1999 ^{B}, v2_2004, Den Haag 2006b)	(*unknown*)	Conformational (partial)	-	[34, 149, 169]
GII4v2.6	IgG1	Mouse	IP injection	GII.4 (v2_2004)	VLP (baculo)	GII.4 (v2_2004)	(*unknown*)	Conformational (partial)	-	[34]
GII.4-1987-G1	IgG2b	Mouse	Oral	GII.4 (1987)	VLP (VEE)	GII.4 (1987 ^{B}, 1997 ^{B}, 2002 ^{B})	P domain (Epitope A)	Conformational	V294, G295, S296, H297, D298, T368, N372, N373	[35, 86, 103]
GII.4-1987-G2	IgG1	Mouse	Oral	GII.4 (1987)	VLP (VEE)	GII.4 (1987 ^{nb}, 1997 ^{nb}, 2002* ^{nb})	(*unknown*)	Conformational	-	[35, 103]
GII.4-1987-G3	IgG1	Mouse	Oral	GII.4 (1987)	VLP (VEE)	GII.4 (1987 ^{nb}, 1997 ^{nb}, 2002* ^{nb})	(*unknown*)	Conformational	-	[35, 103]
GII.4-1987-G4	IgG1	Mouse	Oral	GII.4 (1987)	VLP (VEE)	GII.4 (1987 ^{B}, 1997 ^{B}, 2002 ^{B})	P domain (Epitope A)	Conformational	V294, G295, S296, H297, D298, T368, N372, N373	[35, 103]
GII.4-1987-G5	IgG1	Mouse	Oral	GII.4 (1987)	VLP (VEE)	GII.4 (1987 ^{B}, 1997 ^{B}, 2002 ^{B})	P domain (Epitope A)	Conformational	V294, G295, S296, H297, D298, T368, N372, N373	[35, 103]
GII.4-2006-G1	IgG2b	Mouse	Oral	GII.4 (2006b)	VLP (VEE)	GII.4 (2005*, 2006* ^{nb})	(*unknown*)	Conformational	-	[35]
GII.4-2006-G2	IgG2b	Mouse	Oral	GII.4 (2006b)	VLP (VEE)	GII.4 (2004*, 2006 ^{B}, 2009 ^{B}, 2012* ^{nb}, P.D1* ^{B})	P domain (Epitope A)	Conformational	A294, G295, S296, R297, N298, S368, E372, N373	[35, 37, 38, 86, 103, 150]
GII.4-2006-G3	IgG2a	Mouse	Oral	GII.4 (2006b)	VLP (VEE)	GII.4 (2002 ^{B}, 2004, 2005, 2006 ^{B}, 2008*, 2009 ^{B}, P.D1 ^{B})	P domain (Epitope A)	Conformational	A294, G295, S296, R297, N298, S368, E372, N373	[35, 37, 38, 103, 150]
GII.4-2006-G4	IgG1	Mouse	Oral	GII.4 (2006b)	VLP (VEE)	GII.4 (2005, 2006 ^{B}, 2009 ^{B}, P.D1* ^{B})	P domain (Epitope A)	Conformational	A294, G295, S296, R297, N298, S368, E372, N373	[35, 37, 38, 103, 150]
GII.4-2006-G6	IgG1	Mouse	Oral	GII.4 (2006b)	VLP (VEE)	GII.1*, GII.2*, GII.3*, GII.4 (1997 ^{nb}, 2002 ^{nb}, 2004, 2005, 2006 ^{B}, 2007*, 2008, 2009 ^{B}, 2012* ^{B}, P.D1* ^{B}, GII.14	P domain (Epitope A)	Conformational	A294, G295, S296, R297, N298, S368, E372, N373	[35, 37, 38, 150]

GII.4-2006-G7	IgG1	Mouse	Oral	GII.4 (2006b)	VLP (VEE)	GII.1*, GII.2*, GII.3*, GII.4 (1997* ^{nb}, 2002* ^{nb}, 2004*, 2005, 2006 ^{B}, 2008*, 2009 ^{B}, 2012* ^{B}, P.D1 ^{B}), GII.14*	P domain (Epitope A)	Conformational	A294, G295, S296, R297, N298, S368, E372, N373	[35, 37, 38, 103, 150]
V6-29	IgG	Mouse	IP injection	GI.4 + GII.6	VLP (baculo)	GII.1, GII.2, GII.3, GII.4, GII.5, GII.6, GII.12, GII.13	-	-	-	[195]
212-60-5	IgG	Mouse	IP injection	GI.4 + GII.6	VLP (baculo)	GII.1, GII.3, GII.6, GII.12	-	-	-	[195]
GII.4-2002-G1	IgG2b	Mouse	-	GII.4 (2002)	VLP (VEE)	GII.1*, GII.2*, GII.4 (1987*, 1997*, 2002* ^{nb}, 2004, 2005, 2006*, 2009)	-	Linear	-	[146]
GII.4-2002-G2	IgG2a	Mouse	-	GII.4 (2002)	VLP (VEE)	GII.1, GII.2, GII.4 (1987*, 1997*, 2002* ^{nb}, 2004, 2005, 2006*, 2009)	-	Linear	-	[146]
GII.4-2002-G3	IgG2b	Mouse	-	GII.4 (2002)	VLP (VEE)	GII.1*, GII.2*, GII.4 (1987*, 1997*, 2002* ^{nb}, 2004*, 2005, 2006*, 2009), GII.17 (1978*, 2015*)	-	Linear	(*affected by changes in GII.17 Epitope D*)	[146, 167]
GII.4-2002-G4	IgG2b	Mouse	-	GII.4 (2002)	VLP (VEE)	GII.4 (1987*, 1997, 2002 ^{nb}, 2004, 2005, 2006*, 2009)	-	Linear	-	[146]
GII.4-2002-G5 (GII.4G, MAB227P)	IgG	Mouse	-	GII.4 (2002)	VLP (VEE)	GII.4 (2002, 2006 ^{nb}, 2009 ^{B}, 2012 ^{B}, MC4, MC12)	P domain (Epitope G)	Conformational	(*unknown*)	[37, 86, 98, 146, 154, 160]
GII.4-2002-G6 (GII.4E)	IgG1	Mouse	-	GII.4 (2002)	VLP (VEE)	GII.4 (1987* ^{nb}, 1997* ^{nb}, 2002 ^{B})	P domain (Epitope E)	Conformational	S407, T412, G413	[146, 160]
A3	IgG2b	Mouse	Subcut injection	GII.4 (MD2004-3)	VLP (baculo)	GII.1*, GII.2*, GII.4 (MD2004-3 ^{B}, HS-191, MD145-12)	P domain (Antigenic site I)	Conformational	-	[104]
A6	IgG2a	Mouse	Subcut injection	GII.4 (MD2004-3)	VLP (baculo)	GII.4 (MD2004-3 ^{B}, MD145-12*)	P domain (Antigenic site IIa) (Epitope A)	Conformational	A294, D295, A296, Q297, N298, S368, S372, N373	[104]
A10	IgG1	Mouse	Subcut injection	GII.4 (MD2004-3)	VLP (baculo)	GII.4 (MD2004-3 ^{B})	P domain (Antigenic site IIa) (Epitope A)	Conformational	A294, D295, A296, Q297, N298, S368, S372, N373	[104]
B11	IgG1	Mouse	Subcut injection	GII.4 (MD2004-3)	VLP (baculo)	GII.4 (MD2004-3 ^{B})	P domain (Antigenic site IIb)	Conformational	(*overlaps with site IIa, unaffected by 294, 295*)	[104]
B12	IgG1	Mouse	Subcut injection	GII.4 (MD2004-3)	VLP (baculo)	GII.4 (MD2004-3 ^{B})	P domain (Antigenic site IIb)	Conformational	(*overlaps with site IIa, unaffected by 294, 295*)	[104]
B15	IgG2b	Mouse	Subcut injection	GII.4 (MD2004-3)	VLP (baculo)	GII.4 (MD2004-3, HS-191, MD145-12, CHDC4871, CHDC5191)	S domain Antigenic site III	Conformational	-	[104]
5B18^	IgG, Fab	Mouse	-	GII.4 (NV-445)	VLP	GII.4 (NV-445), GII.10, GII.12	P1 domain	Conformational	(*GII.10 num.*) V433, E496, N530, Y533, T534, L535	[252]
NVB 114	IgG1	Human	Oral - Natural infection (PBMC-generated)	(*unknown*)	Live virus	GII.4 (1987 ^{B}, 1997 ^{B})	P domain (Epitope A)	Conformational	(*GII.4 1987 num.*) V294, G295, S296, H297, D298, T368, N372, N373	[36]
NVB 97	IgG1	Human	Oral - Natural infection (PBMC-generated)	(*unknown*)	Live virus	GII.4 (2004, 2005 ^{B}, 2006 ^{B,H}, 2009 ^{B}, 2015 ^{B}, P.D1 ^{B}, MC4)	P domain (Epitope D)	Conformational	(*GII.4 2009 num.*) D391, S393, T394, T395, P396	[36, 38, 150, 154, 168]
NVB 111	IgG1	Human	Oral - Natural infection (PBMC-generated)	(*unknown*)	Live virus	GII.4 (2006 ^{B,H}, 2009* ^{B}, P.D1 ^{B})	P domain (Epitope A)	Conformational	(*GII.4 2006 num.*) A294, G295, S296, R297, N298, S368, E372, N373	[36, 38, 150]
NVB 43.9	IgG1	Human	Oral - Natural infection (PBMC-generated)	(*unknown*)	Live virus	GII.4 (2006 ^{B,H}, 2009 ^{B}, P.D1* ^{B})	P domain (Epitope A)	Conformational	(*GII.4 2006 num.*) A294, G295, S296, R297, N298, S368, E372, N373	[36, 38, 150, 168]
NVB 37.10	IgG1	Human	Oral - Natural infection (PBMC-generated)	(*unknown*)	Live virus	GII.1, GII.2 (1976, 2002), GII.3, GII.4 (1987 ^{nb}, 1997 ^{nb}, 2002 ^{nb}, 2004, 2005 ^{H}, 2006 ^{H}, 2009 ^{B}), GII.12	(*unknown*)	Conformational	-	[36]
NVB 61.3	IgG1	Human	Oral - Natural infection (PBMC-generated)	(*unknown*)	Live virus	GII.1, GII.2 (1976, 2002), GII.3, GII.4 (1987 ^{nb}, 1997 ^{nb}, 2002 ^{H}, 2004, 2005 ^{nb}, 2006 ^{nb}, 2009 ^{B}), GII.12, GII.17 (1978, 2015)	(*unknown*)	Conformational	(*affected by changes in GII.17 Epitope D*)	[36, 98, 167]
NVB 71.4 (GII.4F)	IgG1, Fab	Human	Oral - Natural infection (PBMC-generated)	(*unknown*)	Live virus	GII.4 (1987 ^{B}, 1997 ^{B}, 2002 ^{B}, 2004, 2005 ^{B,H}, 2006 ^{B,H}, 2009 ^{B}, 2012 ^{B}, P.D1 ^{B}, P.D302 ^{B})	P domain (Epitope F)	Conformational	V327, V404	[36, 38, 98, 150, 160]
MBS223P	-	Mouse	-	GII.4 (2006b)	-	GII.4 (1987, 1997, 2002, 2004, 2005, 2006 ^{nb})	-	Conformational	-	[37]
MBS224P	-	Mouse	-	GII.4 (2006b)	-	GII.4 (2002, 2004, 2005, 2006 ^{nb}, 2009 ^{B})	-	Conformational		[37]
MBS225P	-	Mouse	-	GII.4 (2006b)	-	GII.1, GII.2, GII.4 (1987, 1997, 2002, 2004, 2005, 2006 ^{nb}, 2009 ^{nb})	-	Conformational	-	[37]

MBS226P	-	Mouse	-	GII.4 (2006b)	-	GII.1, GII.2, GII.4 (1987, 1997, 2002, 2004, 2005, 2006 ^{nb}, 2009 ^{nb})	-	Conformational	-	[37]
NO37	IgG	Mouse	Oral	GII.4 (2009)	VLP (VEE)	GII.4 (2005, 2006 ^{B}, 2009 ^{B})	P domain (Epitope A)	Linear	P294, G295, S296, R297, N298, A368, D372, N373	[37, 38, 154]
NO52	IgG	Mouse	Oral	GII.4 (2009)	VLP (VEE)	GII.4 (2005, 2006 ^{B}, 2009 ^{B}, 2012* ^{B}, MC4)	P domain (Epitope A)	Conformational	P294, G295, S296, R297, N298, A368, D372, N373	[37, 38, 154]
NO66	IgG	Mouse	Oral	GII.4 (2009)	VLP (VEE)	GII.4 (2009 ^{B}, 2012 ^{B})	P domain (Epitope D)	Conformational	D391, S393, T394, T395, P396	[37, 38, 154]
NO193	IgG	Mouse	Oral	GII.4 (2009)	VLP (VEE)	GII.4 (2004, 2005, 2006 ^{nb}, 2009 ^{B})	(*unknown*)	Conformational	-	[37]
NO224	IgG	Mouse	Oral	GII.4 (2009)	VLP (VEE)	GII.4 (2009 ^{B}, 2012* ^{B})	(*unknown, near Epitope D*)	Conformational	-	[37, 38, 154]
12A2	scFv	Human	(phage display selection)	GII.4 (r104)	VLP (baculo)	GII.4 (r104 ^{B})	-	-	-	[245]
12A11	scFv	Human	(phage display selection)	GII.4 (r104)	VLP (baculo)	GII.1, GII.3, GII.4 (r104 ^{B}), GII.5, GII.6, GII.7, GII.12, GII.13	-	-	-	[196, 245]
12B10	scFv	Human	(phage display selection)	GII.4 (r104)	VLP (baculo)	GII.1, GII.4(r104 ^{B}), GII.6, GII.7	-	-	-	[245]
1A9	scFv	Human	(phage display selection)	GII.4 (r104)	VLP (baculo)	GII.4 (r104 ^{B})	-	-	-	[245]
5A8	scFv	Human	(phage display selection)	GII.4 (r104)	VLP (baculo)	GII.4 (r104 ^{B})	-	-	-	[245]
SMV37	IgG1 (k)	Mouse	IP injection	GII.2 (1976)	VLP (VEE)	GII.2 (1976 ^{nb}, 2002* ^{nb}, 2008* ^{nb}, 2010* ^{nb})	-	Conformational	-	[107]
SMV59	IgG1 (k)	Mouse	IP injection	GII.2 (1976)	VLP (VEE)	GII.2 (1979), GI.1, GI.3, GII.1, GII.2 (1979 ^{nb}, 2002 ^{nb}, 2008 ^{nb}, 2010 ^{nb}), GII.4 (1987, 1997, 2002, 2004, 2006, 2009, 2012), GII.12	S domain (*put.*)	Conformational	-	[107]
SM114	IgG2b (k)	Mouse	IP injection	GII.2 (1976)	VLP (VEE)	GII.2 (1976 ^{nb}, 2002* ^{nb}, 2008* ^{nb}, 2010* ^{nb})	-	Conformational	-	[107]
SMV129	IgG2a (k)	Mouse	IP injection	GII.2 (1976)	VLP (VEE)	GII.2 (1976 ^{B}, 2002* ^{B}, 2008* ^{B}, 2010* ^{B})	-	Conformational	-	[107]
SMV130	IgG1 (k)	Mouse	IP injection	GII.2 (1976)	VLP (VEE)	GII.2 (1976 ^{nb}, 2002 ^{nb}, 2008 ^{nb}, 2010 ^{nb})	-	Conformational	-	[107]
SMV187	IgG1 (k)	Mouse	IP injection	GII.2 (1976)	VLP (VEE)	GII.2 (1976 ^{B}, 2002 ^{B}, 2008 ^{B}, 2010 ^{B})	-	Conformational	-	[107]
SMV276	IgG2b (k)	Mouse	IP injection	GII.2 (1976)	VLP (VEE)	GII.2 (1976 ^{nb}, 2002 ^{nb}, 2008 ^{nb}, 2010 ^{nb})	-	Conformational	-	[107]
Nano-25	VHH	Alpaca	Subcut injection	GII.10	VLP (baculo)	GII.10 ^{nb}	P domain	Conformational	S256, S429, P432, V433, D479, Q531, F532	[127, 129]
mAb 10E2	-	-	-	GII.4 (Minerva)	VLP	GII.4 (NSW-2012)	P domain	-	-	[41, 127]
mAb 4933	-	Mouse	-	GII.4 (Minerva)	VLP	GII.4 (NSW-2012), GII.10, GII.12	P domain	-	-	[41, 127]
mAb 2H2	-	-	-	GII.4 (Minerva)	VLP	GII.4 (NSW-2012)	P domain	-	-	[41]
mAb 564	-	-	-	GII.4 (Minerva)	VLP	GII.4 (NSW-2012)	P domain	-	-	[41]
mAb 6C10	-	-	-	GII.4 (Minerva)	VLP	GII.4 (NSW-2012)	P domain	-	-	[41]
mAb SM875	-	-	-	GII.4 (Sydney2012)	P domain	GII.4 (NSW-2012)	P domain	-	-	[41]
mAb SM874 -CM355	-	-	-	GII.4 (Saga-2006)	P domain	GII.4 (NSW-2012), GII.17*	P domain	-	-	[41]
M1	VHH	Llama	IM injection	GII.4 (MD2004)	VLP (baculo)	GII.4 (MD2004 ^{B}, CHDC4871, Rockville, MD145 ^{B}, HS191)	P domain	Conformational	(*unique epitope*)	[102]
M2	VHH	Llama	IM injection	GII.4 (MD2004)	VLP (baculo)	GII.4 (Rockville, MD2004 ^{nb}, MD145* ^{nb}, HS191)	P domain	Conformational	-	[102]
M3	VHH	Llama	IM injection	GII.4 (MD2004)	VLP (baculo)	GII.4 (CHDC4871, Rockville, MD2004 ^{B,H}, MD145 ^{B,H}, HS191), GII.6	P domain	Conformational	(*same epitope as M4, M5, M8*)	[102]
M4	VHH	Llama	IM injection	GII.4 (MD2004)	VLP (baculo)	GII.1, GII.2, GII.3 (Toronto, CHDC2005, CHDC5261 CHDC4031, Maizuru2000, Aus2001, Aus2007, Aus2008, CHDC32), GII.4 (CHDC4871, Rockville, MD2004 ^{B,H}, MD145 ^{B,H}, HS191), GII.6, GII.7	P domain	Conformational	(*same epitope as M3, M5, M8*)	[102]
M5	VHH	Llama	IM injection	GII.4 (MD2004)	VLP (baculo)	GII.1, GII.2, GII.3 (Toronto, CHDC2005, CHDC5261 CHDC4031, Maizuru2000, Aus2001, Aus2007, Aus2008, CHDC32), GII.4 (MD145* ^{B,H}, CHDC4871, Rockville, MD2004 ^{B,H}, HS191), GII.6, GII.7	P domain	Conformational	(*same epitope as M3, M4, M8*)	[102]
M6	VHH	Llama	IM injection	GII.4 (MD2004)	VLP (baculo)	GII.1, GII.2, GII.3 (Toronto, CHDC2005, CHDC5261 CHDC4031, Maizuru2000, Aus2001, Aus2007, Aus2008, CHDC32), GII.4 (CHDC4871, Rockville, MD2004 ^{nb}, MD145 ^{H}, HS191), GII.6, GII.7, GII.14	P1 domain	Linear	517-528: ESWVNPFYTLAP	[102]
M7	VHH	Llama	IM injection	GII.4 (MD2004)	VLP (baculo)	GII.4 (MD2004 ^{B})	P domain (Epitope C)	Conformational	G340, E376	[102]
M8	VHH	Llama	IM injection	GII.4 (MD2004)	VLP (baculo)	GII.1, GII.2, GII.3 (Toronto, CHDC2005, CHDC5261 CHDC4031, Maizuru2000, Aus2001, Aus2007,	P domain	Conformational	(*same epitope as M3, M4, M5*)	[102]

						Aus2008, CHDC32), GII.4 (CHDC4871, Rockville, MD2004 ^{H}, MD145* ^{B,H}, HS191), GII.6, GII.7				
2C3G3	IgG, scFv	Mouse	IP injection	GII.4 (2006b)	VLP (baculo)	GII.4 (Den Haag 2006b ^{B}, Apeldoorn 2007)	P domain	Conformational	P245, E247, I389, Q390, R397, R435, G443, Y444, P445, N446, D448	[149, 169]
Nano-4	VHH	Alpaca	Subcut injection	GII.10	VLP (baculo)	GII.1, GII.10 ^{nb}, GII.12, GII.17	P domain (bottom, monomeric)	Conformational	(*GII.17 num.*) R482, T483, E486, D516, N520, Y523, S524, A526	[129]
Nano-14	VHH	Alpaca	Subcut injection	GII.10	VLP (baculo)	GII.10 ^{B}	P domain (top, dimeric)	Conformational	Chain A: H298, R299, E333, V361, A363, W381, E382, D403, K449<br>Chain B: H358, Q384	[129]
Nano-26	VHH	Alpaca	Subcut injection	GII.10	VLP (baculo)	GII.1, GII.2*, GII.4 (Saga 2006*, Sydney2012 ^{B}), GII.10 ^{B}, GII.17	P domain (side/bottom, dimeric)	Conformational	Chain A: I231, P488<br>Chain B: D269, E271, L272, E274, T276, D316, Y470, E471, E472, P475	[129]
Nano-27	VHH	Alpaca	Subcut injection	GII.10	VLP (baculo)	GII.10* ^{nb}	P domain (side/low, monomeric)	Conformational	R484, G491, R492, T493, E496, T534, A536, P537	[129]
Nano-32	VHH	Alpaca	Subcut injection	GII.10	VLP (baculo)	GII.10 ^{B}	P domain (side/middle, dimeric)	Conformational	Chain A: E236, R287, P314, D316, W343, N344<br>Chain B: D247, V248, R492, P518, T519	[129]
Nano-42	VHH	Alpaca	Subcut injection	GII.10	VLP (baculo)	GII.4 (2012 ^{nb}), GII.10 ^{nb}	P domain (bottom, monomeric)	Conformational	D526, W528, V529, N530, F532, T534, A536	[129]
GII.4-2012-G1	-	Mouse	-	GII.4 (2012)	VLP (VEE)	GII.4 (2012 ^{B}, 2015 ^{B})	P domain (Epitope A)	Conformational	T294, G295, S296, R297, N298, E368, D372, R373	[154, 168]
GII.4-2012-G2	-	Mouse	-	GII.4 (2012)	VLP (VEE)	GII.4 (2012 ^{B}, 2015 ^{B})	(*Unknown*)	-	-	[168]
GII.4-2012-G3	-	Mouse	-	GII.4 (2012)	VLP (VEE)	GII.4 (2012 ^{B})	P domain (Epitope A)	Conformational	T294, G295, S296, R297, N298, E368, D372, R373	[168]
GII.4-2012-G5	-	Mouse	-	GII.4 (2012)	VLP (VEE)	GII.4 (2012 ^{B}, 2015 ^{B})	P domain (Epitope A)	Conformational	T294, G295, S296, R297, N298, E368, D372, R373	[154, 168]
GII.4-2012-G6	-	Mouse	-	GII.4 (2012)	VLP (VEE)	GII.4 (2012 ^{B}, 2015 ^{B})	P domain (Epitope A)	Conformational	T294, G295, S296, R297, N298, E368, D372, R373	[168]
GII.4-2012-G7	-	Mouse	-	GII.4 (2012)	VLP (VEE)	GII.4 (2012 ^{B})	P domain (Epitope A)	Conformational	T294, G295, S296, R297, N298, E368, D372, R373	[168]
GII.4-2012-G8	-	Mouse	-	GII.4 (2012)	VLP (VEE)	GII.4 (1987, 1997, 2009, 2012 ^{B})	P domain (Epitope H)	(*unknown*)	(*influenced by Epitope A*)	[168]
NORO-115	IgG (k)	Human	Oral – natural infection (PBMC-generated)	GII.4 (Sydney2012)	Live virus	GII.4 (HOV ^{B}, Sydney2012 ^{B,H})	P domain (Group 1)	-	-	[118]
NORO-313.1	IgG (k)	Human	Oral – natural infection (PBMC-generated)	GII.4 (Sydney2012)	Live virus	GII.4 (HOV ^{B}, Sydney2012 ^{B,H})	P domain (Group 2)	-	-	[118]
NORO-246A	IgG (l)	Human	Oral – natural infection (PBMC-generated)	GII.4 (Sydney2012)	Live virus	GII.4 (HOV ^{B}, Sydney2012 ^{B,H})	P domain (Group 2)	-	-	[118]
NORO-250B	IgG (l)	Human	Oral – natural infection (PBMC-generated)	GII.4 (Sydney2012)	Live virus	GII.4 (HOV ^{B}, Sydney2012 ^{B,H,N})	P domain (Group 3)	-	-	[118]
NORO-279A	IgG (l)	Human	Oral – natural infection (PBMC-generated)	GII.4 (Sydney2012)	Live virus	GII.4 (HOV ^{nb}, Sydney2012 ^{nb})	S domain	-	-	[118]
NORO-329A	IgG (l)	Human	Oral – natural infection (PBMC-generated)	GII.4 (Sydney2012)	Live virus	GII.4 (HOV ^{B}, Sydney2012 ^{B,H})	S + P domain (Group 3)	Conformational (quaternary structure)	-	[118]
NORO-118	IgG (l)	Human	Oral – natural infection (PBMC-generated)	GII.4 (Sydney2012)	Live virus	GII.4 (HOV ^{B}, Sydney2012 ^{H})	P domain	-	-	[118]
NORO-316	IgG (l)	Human	Oral – natural infection (PBMC-generated)	GII.4 (Sydney2012)	Live virus	GII.4 (HOV ^{B}, Sydney2012 ^{B,H})	P domain (Group 3)	-	-	[118]

NORO-202A.1	IgG (l)	Human	Oral – natural infection (PBMC-generated)	GII.4 (Sydney2012)	Live virus	GII.4 (HOV [nb], Sydney2012 [nb])	S domain	-	-	[118]
NORO-312A	IgG (l)	Human	Oral – natural infection (PBMC-generated)	GII.4 (Sydney2012)	Live virus	GII.4 (HOV [B], Sydney2012 [B,H])	S + P domain (Group 3)	Conformational (quaternary structure)	-	[118]
NORO-317	IgG (l)	Human	Oral – natural infection (PBMC-generated)	GII.4 (Sydney2012)	Live virus	GII.4 (HOV [B], Sydney2012 [B,H])	P domain (Group 3)	-	-	[118]
NORO-303	IgG (l)	Human	Oral – natural infection (PBMC-generated)	GII.4 (Sydney2012)	Live virus	GII.4 (HOV [nb], Sydney2012 [nb])	P domain (Group 1)	-	-	[118]
NORO-263	IgG (l)	Human	Oral – natural infection (PBMC-generated)	GII.4 (Sydney2012)	Live virus	GII.4 (HOV [B], Sydney2012 [B,N])	P domain (Group 2)	-	-	[118]
NORO-296A	IgG (l)	Human	Oral – natural infection (PBMC-generated)	GII.4 (Sydney2012)	Live virus	GII.4 (HOV [B], Sydney2012 [B,H])	S + P domain (Group 3)	Conformational (quaternary structure)	-	[118]
NORO-327A	IgG (l)	Human	Oral – natural infection (PBMC-generated)	GII.4 (Sydney2012)	Live virus	GII.4 (HOV [B], Sydney2012 [B,H])	P domain (Group 3)	-	-	[118]
NORO-315B	IgG (l)	Human	Oral – natural infection (PBMC-generated)	GII.4 (Sydney2012)	Live virus	GII.4 (HOV [B], Sydney2012 [B,H])	P domain (Group 3)	-	-	[118]
NORO-251A	IgG (l)	Human	Oral – natural infection (PBMC-generated)	GII.4 (Sydney2012)	Live virus	GII.4 (HOV [nb], Sydney2012 [nb])	S domain	-	-	[118]
NORO-256A	IgG (l)	Human	Oral – natural infection (PBMC-generated)	GII.4 (Sydney2012)	Live virus	GII.4 (HOV [nb], Sydney2012 [nb])	S domain	-	-	[118]
NORO-278	IgG (l)	Human	Oral – natural infection (PBMC-generated)	GII.4 (Sydney2012)	Live virus	GII.4 (HOV [nb], Sydney2012 [nb])	P domain	-	-	[118]
NORO-123	IgG (l)	Human	Oral – natural infection (PBMC-generated)	GII.4 (Sydney2012)	Live virus	GII.4 (HOV [B], Sydney2012 [B,H])	P domain (Group 1)	-	-	[118]
NORO-310A	IgG (k)	Human	Oral – natural infection (PBMC-generated)	GII.4 (Sydney2012)	Live virus	GII.4 (HOV [nb], Sydney2012 [nb])	S domain	-	-	[118]
NORO-318	IgA (k)	Human	Oral – natural infection (PBMC-generated)	GII.4 (Sydney2012)	Live virus	GII.4 (HOV [B], Sydney2012 [B,H,N])	P domain (Group 2 + 3)	-	-	[118]
NORO-320	IgA (k)	Human	Oral – natural infection (PBMC-generated)	GII.4 (Sydney2012)	Live virus	GII.4 (HOV [B], Sydney2012 [B,H,N])	P domain (Group 1)	-	-	[118]
NORO-273A	IgA (k)	Human	Oral – natural infection (PBMC-generated)	GII.4 (Sydney2012)	Live virus	GII.4 (HOV [B], Sydney2012 [B,H,N])	P domain (Group 1)	-	-	[118]
NORO-232A.2	IgA (k)	Human	Oral – natural infection (PBMC-generated)	GII.4 (Sydney2012)	Live virus	GII.4 (HOV [B], Sydney2012 [B,H])	S + P domain (Group 2)	Conformational (quaternary structure)	-	[118]
Mab #1	IgG	Mouse	-	GII.4 (Minerva)	VLP (baculo)	GII.4 (CHDC-1974, Saga-2006)	-	-	-	[119]
Mab #2	IgG	Mouse	-	GII.4 (Minerva)	VLP (baculo)	GII.4 (Saga-2006, NSW-2012)	-	-	-	[119]
Mab #3	IgG	Mouse	-	GII.4 (Minerva)	VLP (baculo)	GII.4 (CHDC-1974, Saga-2006, NSW-2012)	-	-	-	[119]
Mab #4	IgG	Mouse	-	GII.4 (Minerva)	VLP (baculo)	GII.4 (CHDC-1974, Saga-2006, NSW-2012)	-	-	-	[119]
Mab #5	IgG	Mouse	-	GII.4 (Minerva)	VLP (baculo)	GII.4 (CHDC-1974, Saga-2006, NSW-2012)	-	-	-	[119]
Mab #6	IgG	Mouse	-	GII.4 (Minerva)	VLP (baculo)	GII.4 (CHDC-1974, Saga-2006, NSW-2012)	-	-	-	[119]
Mab #7 (10E9)	IgG, Fab	Mouse	-	GII.4 (Minerva)	VLP (baculo)	GII.4 (Saga-2006 [B], NSW-2012 [nb], TCH12-580 [N], Mannheim 2018 [N])	P domain (overlaps HBGA binding epitope)	Conformational	Chain A: F250, D391, T394, T395, R397, R435, Y444, N446, D448, Q504, D506<br>Chain B: T340, D341, S343, R345	[119]
Mab #8	IgG	Mouse	-	GII.4 (Minerva)	VLP (baculo)	GII.4 (Saga-2006, NSW-2012)	-	-	-	[119]

Mab #9	IgG	Mouse	-	GII.4 (Minerva)	VLP (baculo)	GII.4 (CHDC-1974, Saga-2006, NSW-2012)	-	-	-	[119]
Mab #10	IgG	Mouse	-	GII.4 (Minerva)	VLP (baculo)	GII.4 (Saga-2006)	-	-	-	[119]
Mab #11	IgG	Mouse	-	GII.4 (Minerva)	VLP (baculo)	GII.4 (CHDC-1974, Saga-2006, NSW-2012)	-	-	-	[119]

Table S3. mAbs that recognize VLPs across genogroups.

Antibody	Isotype	Source	Initial mode of immunization	Immunogen genotype (Strain)	Immunogen (Origin)	Recognizes (Strain)	Reactive region (Epitope)	Epitope arrangement	Amino acid region	Ref(s)
NV3901	IgG1	Mouse	IP injection	GI.1	VLP (baculo)	GI.1 ^{nb}, GI.2, GI.3, GI.4, GI.6, GI.8, GII.7*, GII.17*, GIV.1*	P1 domain	Linear (*could be partially conformational*)	Between 454-520 Includes E472, K514 (*enhanced by H460, P515*)	[71, 92, 100, 106, 124, 240, 241, 243, 253, 254]
NV3912	IgG1	Mouse	IP injection	GI.1	VLP (baculo)	GI.1 (NV ^{nb}, SEV), GI.2, GI.4, GI.6, GI.7*, GI.8, GII.7*, GII.12*, GII.17*	P1 domain	Linear (*could be partially conformational*)	Between 454-520 Includes E472, K514 (*enhanced by H460, P515*)	[71, 92, 100, 106, 133, 176, 240-243, 247, 255]
NV8812	IgG1	Mouse	IP injection	GI.1	VLP (baculo)	GI.1 ^{H}, GII.7*	P2 domain	Conformational	Between 300-384	[71, 92, 100, 124, 240, 241, 243]
1B4	IgG2b (k)	Mouse	Subcut injection	GII.3	VP1 (bacteria)	GI.1 *, GI.2, GI.3, GI.6, GII.1, GII.2, GII.3, GII.4 (LD), GII.5, GII.6, GII.8, GII.12, GII.17	S domain	Linear	44-54: QQNIIDPWIMN	[244, 249, 256]
1F6	IgG1 (k)	Mouse	Subcut injection	GII.3	VP1 (bacteria)	GI.1, GI.3, GI.4, GI.6, GII.1, GII.2*, GII.3, GII,4 (LD), GII.5, GII.6, GII.8, GII.12, GII.17	S domain	Linear	44-54: QQNIIDPWIMN	[244, 249, 256]
3E7	IgG (k)	Mouse	Subcut injection	GI.1	VP1 (bacteria)	GI.1, GI.4, GII.3*	S domain	Linear	Between 1-43	[244]
NV23	IgG1	Mouse	Oral	GI.1	VLP (baculo)	GI.1 (NV, SEV), GI.2, GI.4, GI.5*, GI.6, GI.7*, GI.8, GI.9, GI. GII.1, GII.2 (SMV, 2002), GII.3, GII.4 (GV*, NAV, HOV, FH, NO, Sydney2012), GII.6*, GII.7*, GII.8, GII.9, GII.12, GII.13, GII.15*, GII.17, GII.21*, GIV.1	P1 domain	Linear	(*GI.1 NV num.*) 437-457: LPQEYISHLASEQAPTVGEA	[100, 134, 242, 253]
NV37^	IgG1	Mouse	Oral	GI.1	VLP (baculo)	GI.1 (NV, SEV), GI.2*, GI.4*, GI.6*, GI.7, GI.8*, GII.1, GII.2*, GII.3*, GII.4 (GV*, NAV*, HOV*, Sydney2012*), GII.6*, GII.7, GII.9*, GII.12* , GII.13, GII.17, GIV.1*	P1 domain	Linear	(*GII.4 HOV num.*) 453-495	[100, 242, 253]
NV3^	IgG1	Mouse	Oral	GI.1	VLP (baculo)	GI.1 (NV, SEV), GI.2*, GI.4*, GI.6*, GI.7, GI.8*, GII.1*, GII.2*, GII.3*, GII.4 (GV*, HOV*, Sydney2012*), GII.6*, GII.7, GII.9*, GII.12, GII.13, GII.17, GIV.1*	P1 domain	Linear	(*GII.4 HOV num.*) 453-495	[100, 242, 253]
NS46	IgG1	Mouse	Oral	GI.1 + GII.2	VLP (baculo)	GII.1, GII.2*, GII.3, GII.4 (GV, NAV, HOV, Sydney2012), GII.6*, GII.7, GII.9, GII.12, GII.13, GII.17, GIV.1*	-	Linear	-	[100, 242]
NS14	IgG1	Mouse	Oral	GI.1 + GII.2	VLP (baculo)	GI.1*, GI.4, GII.1, GII.2, GII.3, GII.4 (GV, NAV, HOV, Sydney2012), GII.6*, GII.7, GII.9, GII.12, GII.13, GII.17, GIV.1*	P1 domain	Linear	(*GII.4 HOV num.*) 473-495 (*enhanced by 453-473*)	[100, 106, 133, 176, 180, 242, 253, 254, 257]
F8^	IgG1	Mouse	Oral	GII.13	VLP (baculo)	GI.1 (NV, SEV), GI.2, GI.4, GI.6, GI.7, GII.2, GII.3, GII.4 (GV, NAV), GII.6, GII.7, GII.12, GII.13, GII.17	P1 domain	Linear	(*GII.4 HOV num.*) 453-495	[242, 253]
F120^	IgG1	Mouse	Oral	GII.13	VLP (baculo)	GI.1 (NV, SEV), GI.2, GI.4, GI.6, GI.7, GII.2, GII.3, GII.4 (GV, NAV), GII.6, GII.7, GII.12, GII.13, GII.17	P1 domain	Linear	(GI.1 NV num.) 437-457: LPQEYISHLASEQAPTVGEA	[242, 253]
CM39	IgG	Mouse	IP injection	GIII.1	VLP (baculo)	GII.3*, GIII.1, GIII.2	S domain	Linear	P31, T32, A33, G34, A35, Q36, I37, A38, A39	[115]
CM54	IgM	Mouse	IP injection	GI.2	VLP (baculo)	GI.1, GI.2, GI.3, GI.5, GIII.2	S domain	Linear	L162, E163, D164, V165, R166, N167 (*enhanced by 132-137*)	[114]
MAb14-1	IgG1	Mouse	(added to myeloma cells)	GII.4 (r1207)	VLP (baculo)	GI.1*, GI.3*, GI.4*, GI.6*, GII.1, GII.2, GII.3, GII.4, GII.5, GII.6, GII.7, GII.12, GII.13, GII.14, GII.16	P1 domain	Conformational	418-426: APAVAPTFP 526-534: LAPMGNGAG	[258]
N2C3	IgG2a	Mouse	IP injection	GII.4 (NVgz01)	VLP	GI.1, GI.2, GI.4, GI.5, GI.6, GI.7*, GI.8*, GII.1, GII.2, GII.3*, GII.4 (NVgz01), GII.7, GII.8, GII.12*, GII.14, GII.16*, GII.21, GIII.1*, GIII.2*, GIV, GV	S domain	Linear	W55, I56, R57, N58, N59, F60	[116, 251]
GII.4-1987-M1	IgM	Mouse	Oral	GII.4 (1987)	VLP (VEE)	GI.1*, GI.2*, GI.3*, GI.4*, GII.1, GII.4 (1987* ^{nb}, 1997*, 2002, 2004*, 2005*), GII.14*	-	-	-	[35]
GII.4-1987-M2	IgM	Mouse	Oral	GII.4 (1987)	VLP (VEE)	GI.1, GI.2, GI.3, GI4, GII.1, GII.2, GII.3, GII.4 (1987 ^{nb}, 1997, 2002, 2004, 2005, 2006), GII.14	-	-	-	[35]
M9-110	IgG	Mouse	IP injection	GI.4 + GII.6	VLP (baculo)	GI.1, GI.3, GI.4, GI.7, GI.8, GI.12, GII.1, GII.2, GII.3, GII.4, GII.5, GII.6, GII.12, GII.13	-	-	-	[195]
TV20	IgG	Mouse	IP injection	GII.3	VLP (baculo)	GI.1, GI.3, GII.1, GII.2, GII.3, GII.4 (MD2004-3 ^{nb}), GIV.1, GV	S domain	Linear	I52, D53, P54, W55, I56	[132, 259]

CV-1A5	scFv	Human	(phage display selection)	GI.4	VLP (baculo)	GI.1*, GI.2*, GI.3*, GI.4 [B], GI.6*, GII.1, GII.3, GII.4 (r104* [nb]), GII.5*, GII.6*, GII.7*, GII.12, GII.13	-	-	-	[245]
HJT-R3-A9^	scFv	Human	(phage display selection)	GII.4 (HOV)	VLP	GI.1, GI.2, GI.4, GI.5*, GI.6*, GI.7*, GI.8, GI.9, GII.1, GII.2 (SMV, 2002), GII.3, GII.4 (GV*, HOV, FH, NO, Sydney2012), GII.5, GII.6*, GII.7*, GII.8, GII.9, GII.12, GII.13, GII.15*, GII.17, GII.21*, GIV.1*	S domain	Linear	-	[134, 176]
HJT-R3-F7	scFv	Human	(phage display selection)	GII.4 (HOV)	VLP	GI.1*, GI.6*, GI.7*, GII.2*, GII.3*, GII.4 (HOV*), GII.6*, GII.7*, GII.12*, GII.17*	P1 domain	Linear	Between 417-488 (473-488)	[176]
HJL-R3-B4	scFv	Human	(phage display selection)	GII.4 (HOV)	VLP	GI.1, GI.6, GI.7, GII.4 (HOV), GII.7*, GII.12*	-	-	-	[176]
HJL-R3-D11	scFv	Human	(phage display selection)	GII.4 (HOV)	VLP	GI.1*, GI.6*, GI.7*, GII.2, GII.3, GII.4 (HOV), GII.6*, GII.7, GII.12, GII.17	-	-	-	[176]
HJL-R3-F11	scFv	Human	(phage display selection)	GII.4 (HOV)	VLP	GII.4 (HOV)	-	-	-	[176]
HJL-R3-G8	scFv	Human	(phage display selection)	GII.4 (HOV)	VLP	GII.4 (HOV)	-	-	-	[176]
HJL-R3-H1	scFv	Human	(phage display selection)	GII.4 (HOV)	VLP	GII.4 (HOV)	-	-	-	[176]
HJL-R3-D1	scFv	Human	(phage display selection)	GII.4 (HOV)	VLP	GII.4 (HOV)	-	-	-	[176]
HJL-R3-E4	scFv	Human	(phage display selection)	GII.4 (HOV)	VLP	GII.4 (HOV)	-	-	-	[176]
5C4.10 (5C4)	IgG2b	Mouse	Oral	GV (MNV-1)	Live virus	GI.1, GII.4 (Saga-2006, Sydney2012*), GII.10*, GII.12*, GV (MNV-1 [nn], WU20 [nn], CR1 [nn], CR3 [nn], CR6 [nn], CR7 [nn], MNV3 [nn], MNV-4 [nn], S99 [nn], WU11 [nn])	S domain	Linear	-	[65, 161]
NV57^	IgG	Mouse	Oral	GI.1	VLP (baculo)	GI.1*, GI.2*, GI.4*, GI.6*, GII.1*, GII.2*, GII.3, GII.4 (GV, HOV, Sydney2012), GII.6*, GII.7, GII.9, GII.12, GII.17, GIV.1*	P1 domain	Linear	(*GII.4 HOV num.*) 453-495	[100, 253]
NS22	IgG	Mouse	Oral	GI.1 + GII.2	VLP (baculo)	GI.1, GI.2, GI.4, GI.6, GI.7, GI.8, GII.1, GII.2, GII.3, GII.4 (GV, HOV, Sydney2012), GII.6*, GII.7, GII.9, GII.12, GII.17, GIV.1	P1 domain	Linear	(*GI.1 NV num.*) 437-457: LPQEYISHLASEQAPTVGEA	[100, 253]
NV7^	-	Mouse	Oral	GI.1	VLP (baculo)	GI.1, GII.2, GII.3, GII.4, GII.6, GII.12, GII.17	P1 domain	Linear	(*GII.4 HOV num.*) 453-495	[253]
NS941^	-	Mouse	Oral	GI.1 + GII.2	VLP (baculo)	GI.4, GII.6, GII.12	P1 domain	Linear	(*GII.4 HOV num.*) 453-495	[253]
Nano-85	VHH	Alpaca	Subcut injection	GII.10	VLP (baculo)	GI.1*, GII.4 (Saga-2006 [B], NSW-2012, Sydney2012 [B]), GII.10 [B], GII.12, GII.17	P domain (bottom, monomeric)	Conformational	L477, F525, W528, V529, N530, F532, Y533, T534	[127-129]
mAb NV7	-	Mouse	Subcut injection	GI.1	VLP (baculo)	GI.1 [H], GI.3, GII.1, GII.2, GII.4 (MD2004-3), GIV.1	P domain – Antigenic Site IV	Conformational	-	[132]
mAb NV81	-	Mouse	Subcut injection	GI.1	VLP (baculo)	GI.1 [nb], GI.3, GII.1, GII.2, GII.4 (MD2004-3), GIV.1	P domain – Antigenic Site III	Conformational	-	[132]
10B11	IgG	Mouse	IM and ID injection	GI.2 + GI.7 + GII.2 + GII.3 + GII.4 + GII.6 + GII.7 + GII.17	VLP (baculo)	GI.2, GI.7*, GII.2*, GII.3, GII.4*, GII.6, GII.7, GII.17	-	-	-	[260]
8D8	IgG	Mouse	IM and ID injection	GI.2 + GI.7 + GII.2 + GII.3 + GII.4 + GII.6 + GII.7 + GII.17	VLP (baculo)	GI.2, GI.7, GII.2, GII.3, GII.4, GII.6, GII.7, GII.17	S domain	Linear	(*GII.3 num.*) 45-56: QNIIDPWIMNNF	[260]

Table S4. mAbs that are reactive to GV strains.

Antibody	Isotype	Source	Initial mode of immunization	Immunogen genotype (Strain)	Immunogen (Origin)	Recognizes (Strain)	Reactive region (Epitope)	Epitope arrangement	Amino acid region	Ref(s)
MAb A6.2 (MAb A6.2.1)	IgG2a, Fab	Mouse	IP injection	GV (MNV-1)	Live virus	GV (MNV-1 ^{N}, CW.1 P3 ^{N}, CW3, CR1 ^{N}, CR3 ^{N}, CR6* ^{nn}, CR7* ^{nn}, MNV3* ^{nn}, MNV4 ^{N}, S99 ^{N}, WU11* ^{nn}, WU20* ^{nn})	P2 domain (*Overlaps CD300lf binding site*)	Conformational (*Partially linear?*)	Loops A'-B' - 294-303<br>Loops E'-F'- 379-388<br>(*escape at V378, A382, D385, L386*)	[45, 49, 65, 161, 162, 172, 174, 175, 261]
MAb A6.1	IgG2a	Mouse	IP injection	GV (MNV-1)	Live virus	GV (MNV-1 ^{N})	-	-	-	[45]
MAb H6.1	IgG2a	Mouse	IP injection	GV (MNV-1)	Live virus	GV (MNV-1 ^{N})	-	-	-	[45]
2D3.7 (2D3)	IgA, Fab	Mouse	Oral	GV(MNV-1)	Live virus	GV (MNV-1* ^{N}, CR1 ^{N}, CR3 ^{N}, CR6* ^{N}, CR7* ^{N}, MNV3* ^{N}, MNV4 ^{N}, S99 ^{N}, WU11* ^{N}, WU20* ^{N})	P domain (*Overlaps CD300lf binding site [put.]*)	Conformational	Loops A'-B', E'-F' (*influenced by V339, D348*)	[135, 161, 172]
2D3.9	IgA	Mouse	Oral	GV(MNV-1)	Live virus	GV (MNV-1 ^{N}, WU20 ^{N})	P domain	Conformational	-	[65]
3C7.14	IgG1	Mouse	Oral	GV(MNV-1)	Live virus	GV (MNV-1 ^{nn}, WU20 ^{nn})	(*unknown*)	Linear	-	[65]
3C7.16	IgG1	Mouse	Oral	GV(MNV-1)	Live virus	GV (MNV-1 ^{nn}, WU20* ^{nn})	(*unknown*)	Linear	-	[65]
4F9.3	IgA	Mouse	Oral	GV(MNV-1)	Live virus	GV (MNV-1 ^{N}, WU20* ^{N})	(*unknown*)	Conformational	-	[65]
4F9.4 (4F9)	IgA	Mouse	Oral	GV(MNV-1)	Live virus	GV (MNV-1* ^{N}, CR1 ^{N}, CR1 ^{N}, CR3* ^{N}, CR6* ^{N}, CR7* ^{nn}, MNV3* ^{N}, MNV4* ^{N}, S99 ^{N}, WU11* ^{nn}, WU20* ^{N})	P domain	Linear	(*influenced by V339, D348*)	[65, 161]
5C4.7	IgG2b	Mouse	Oral	GV(MNV-1)	Live virus	GV (MNV-1 ^{nn}, WU20 ^{nn})	S domain	Linear	-	[65]
9B4.5	IgG2b	Mouse	Oral	GV(MNV-1)	Live virus	GV (MNV-1* ^{nn})	(*unknown*)	Conformational	-	[65]
2G7.6	IgG1	Mouse	Oral	GV(WU20)	Live virus	GV (MNV-1 ^{nb}, WU20 ^{nb})	(*unknown*)	Linear	-	[65]
2G7.12	IgG1	Mouse	Oral	GV(WU20)	Live virus	GV (MNV-1 ^{nb}, WU20 ^{nb})	(*unknown*)	Linear	-	[65]
3F9.9.15	IgG2a	Mouse	Oral	GV(WU20)	Live virus	GV (MNV-1 ^{nn}, WU20* ^{nn})	P domain	Conformational	-	[65]
3F9.9.20	IgG2a	Mouse	Oral	GV(WU20)	Live virus	GV (MNV-1 ^{nn}, WU20* ^{nn})	P domain	Conformational	-	[65]
4B7.3	IgG2a	Mouse	Oral	GV(WU20)	Live virus	GV (MNV-1 ^{nn}, WU20* ^{nn})	(*unknown*)	Linear	-	[65]
4B7.10	IgG2a	Mouse	Oral	GV(WU20)	Live virus	GV (MNV-1 ^{nn}, WU20* ^{nn})	(*unknown*)	Linear	-	[65]
5C2.6	IgG1	Mouse	Oral	GV(WU20)	Live virus	GV (MNV-1 ^{nn}, WU20 ^{nn})	(*unknown*)	Linear	-	[65]
5C2.9	IgG1	Mouse	Oral	GV(WU20)	Live virus	GV (MNV-1 ^{nn}, WU20 ^{nn})	(*unknown*)	Linear	-	[65]
5D9.19	IgG1	Mouse	Oral	GV(WU20)	Live virus	GV (MNV-1 ^{nn}, WU20 ^{nn})	(*unknown*)	Linear	-	[65]
5D9.5	IgG1	Mouse	Oral	GV(WU20)	Live virus	GV (MNV-1 ^{nn}, WU20 ^{nn})	(*unknown*)	Linear	-	[65]

Table S5. Norovirus T cell epitopes.

MHC Source (Allele)	T cell restriction	Cytokine stimulation	Priming immunogen(s)	Initial mode of immunization	Splenocyte stimulation	Reactive region	Amino acid region	Amino acid sequence	Stimulatory activity confirmed against	Ref(s)
Mouse	CD4+	IFN-γ secretion	MNV-03 (live virus)	Oral	Various VLPs and GI.1 (NV) peptides	VP1 – S domain	83-94	LSLGPHLNPFLL	GI.1 (NV, WC-02)	[185]
Mouse	CD4+	IFN-γ secretion	MNV-03 (live virus)	Oral	Various VLPs and GII.4-2002 peptides	VP1 – P1 domain	461-473	CLLPQEWVQHFYQ	GII.1*, GII.4 (FH-02, LV-87, LV-97, Hu-04, Sak-05*), GII.14*	[185]
Mouse	CD4+	IFN-γ secretion	MNV-03 (live virus)	Oral	Various VLPs and GII.4-2002 peptides	VP1 – P1 domain	471-485	FYQEAAPAQSDVALL	GII.4 (FH-02, LV-97*, Hu-04*)	[185]
Mouse	CD4+	IFN-γ, TNF-α, IL-2 secretion	GII.4 VA387 (VLPs, P dimers, P particles)	Intranasal	GII.4 VA387 peptide	VP1 – P1 domain	235-248	FYQEAAPAQSDVAL	GII.4 (VA387)	[113]
Mouse	CD8+	IFN-γ and TNF-α secretion	MNV-CR6 (live virus)	IV injection	MNV-CR6 peptides	ORF1 - NTPase	424-441	ECQLENQLTAMLRDRNAG	GV (MNV-CR6*)	[58]
Mouse	CD8+	IFN-γ and TNF-α secretion	MNV-CR6 (live virus)	IV injection	MNV-CR6 peptides	VP1 – P2 domain	388-405	DGRVRAVPRSIYSFQDEL	GV (MNV-CR6*)	[58]
Mouse	CD8+	IFN-γ and TNF-α secretion	MNV-CR6 (live virus)	IV injection	MNV-CR6 peptides	VP1 – P1 domain	424-441	LPGEVLLRFRTYMRQLDT	GV (MNV-CR6*)	[58]
Mouse (H-2kb)	CD8+	IFN-γ and TNF-α secretion	MNV-CR6 (live virus)	IV injection	MNV-CR6 peptides	VP1 – P1 domain	519-527	SWVPRLYQL	GV (MNV-CR6, MNV-CW3)	[58]
Mouse	CD4+	IFN-γ secretion	GII.4 or GII.12 (VLPs)	IM injection	GII.4-1999 peptides	VP1 – S domain	22-39	VNNEVMALEPVVGAAIAA	GII.4 (1999, NO, Sydney2012)	[177]
Mouse	CD4+	IFN-γ secretion	GII.4 or GII.12 (VLPs)	IM injection	GII.4-1999 peptides	VP1 – S domain	176-193	IKLIAMLYTPLRANNAGD	GII.4 (1999, NO, Sydney2012)	[177]
Mouse	CD8+	IFN-γ secretion	GII.4 or GII.12 (VLPs)	IM injection	GII.4-1999 peptides	VP1 – P2 domain	309-326	NNYDPTEEIPAPLGTPDF	GII.4 (1999, NO, Sydney2012), GII.12	[177, 262]
Mouse	CD8+	IFN-γ secretion	GII.4 or GII.12 (VLPs)	IM injection	GII.4-1999 peptides	VP1 – P2 domain	316-333	EIPAPLGTPDFVGKIQGL	GII.4 (1999, NO, Sydney2012), GII.12	[177]
Mouse	CD4+	IFN-γ secretion	GII.4 or GII.12 (VLPs)	IM injection	GII.4-1999 peptides	VP1 – P2 domain	337-354	TTREDGSTRAHKATVSTG	GII.4 (1999)	[177]
Mouse	CD4+	IFN-γ secretion	GII.4 or GII.12 (VLPs)	IM injection	GII.4-1999 peptides	VP1 – P2 domain	344-361	TRAHKATVSTGSVHFTPK	GII.4 (1999)	[177, 262]
Mouse	CD8+	IFN-γ secretion	GII.4 or GII.12 (VLPs)	IM injection	GII.4-1999 peptides	VP1 – P1 domain	449-466	DCLLPQEWVQHFYQEAAP	GII.4 (1999)	[177]
Human	(*unknown*)	IFN-γ secretion	Natural infection (PBMC isolation)	Oral	GII.4-1999 peptides	VP1 – S domain	106-123 (*put.*)	QVILAGNAFTAGKIIFAA	-	[81]
Human	(*unknown*)	IFN-γ secretion	Natural infection (PBMC isolation)	Oral	GII.4-1999 peptides	VP1 – S/P domain (spans hinge)	211-228 (*put.*)	DFIFLVPPTVESRTKPFT	-	[81]
Human (HLA-A*02:01)	CD8+	IFN-γ, TNF-α, IL-2 secretion	Natural infection (PBMC isolation)	Oral	Various VLPs and GII.4-1999 peptides	VP1 – S domain	139-148	TMFPHIIVDV	GI.1, GI.3, GII.1, GII.2, GII.3*, GII.4 (1999, FH-02, Apeldoorn 2003, Hu-04, NO-09, Sydney2012), GII.10, GII.12, GII.17	[81, 188]

SUPPLEMENTARY TABLE FOOTNOTES

Tables S1–4

Monoclonal antibodies (mAbs) within the tables are time-ordered and list the earliest characterized mAb of each subset first and the most recently generated mAb last. mAbs marked with (^) have reported variable recognition activity to certain genotypes of norovirus due to binding a masked or conformationally-hidden epitope. The reactive genotypes were updated to reflect current genotyping criteria by cross-checking provided sequences with the RIVM online genotyping tool https://www.rivm.nl/mpf/typingtool/norovirus. Genotypes and strains marked with an asterisk (*) were reported to react weakly with the mAb. Superscript "B" denotes reported HBGA blockade activity, superscript "H" denotes reported hemagglutination inhibition activity (HAI), superscript "N" denotes reported neutralization activity either in vitro or in vivo, and superscripts "nb" and "nn" indicate the mAbs failed to block or neutralize, respectively. Amino acids that are underlined indicate residues that were determined to be essential for mAb binding. References for each mAb include reports of isolation, characterization, and representative applications.

Table S5

VLP immunization or viral infection was followed by T cell isolation and stimulation with overlapping peptides spanning a norovirus genotype of interest. Changes in cytokine release are recorded here as well as their T cell restriction (CD4+ or CD8+). Minimal binding regions and anchor residues are underlined. Weak reactivity is denoted with an asterisk by the genotype and strain tested. Though many of these sequences have been reported to be highly conserved across norovirus genotypes, only three have been verified to induce cross-genotypic T cell responses in vitro (bolded epitopes).

1. Patel, M.M.; Widdowson, M.A.; Glass, R.I.; Akazawa, K.; Vinje, J.; Parashar, U.D. Systematic literature review of role of noroviruses in sporadic gastroenteritis. *Emerg. Infect. Dis.* **2008**, *14*, 1224–1231.
2. Hoa Tran, T.N.; Trainor, E.; Nakagomi, T.; Cunliffe, N.A.; Nakagomi, O. Molecular epidemiology of noroviruses associated with acute sporadic gastroenteritis in children: Global distribution of genogroups, genotypes and GII.4 variants. *J. Clin. Virol.* **2013**, *56*, 185–193.
3. Moore, M.D.; Goulter, R.M.; Jaykus, L.A. Human norovirus as a foodborne pathogen: Challenges and developments. *Annu. Rev. Food Sci. Technol.* **2015**, *6*, 411–433.
4. Dolin, R.; Blacklow, N.R.; DuPont, H.; Buscho, R.F.; Wyatt, R.G.; Kasel, J.A.; Hornick, R.; Chanock, R.M. Biological properties of Norwalk agent of acute infectious nonbacterial gastroenteritis. *Proc. Soc. Exp. Biol. Med.* **1972**, *140*, 578–583.
5. Cutler, A.J.; Oliveira, J.; Ferreira, R.C.; Challis, B.; Walker, N.M.; Caddy, S.; Lu, J.; Stevens, H.E.; Smyth, D.J.; Pekalski, M.L.; et al. Capturing the systemic immune signature of a norovirus infection: An n-of-1 case study within a clinical trial—Wellcome Open Research. *Wellcome Open Res.* **2017**, *2*, 28, doi:10.12688/wellcomeopenres.11300.1.
6. Saito, M.; Goel-Apaza, S.; Espetia, S.; Velasquez, D.; Cabrera, L.; Loli, S.; Crabtree, J.E.; Black, R.E.; Kosek, M.; Checkley, W.; et al. Multiple norovirus infections in a birth cohort in a Peruvian Periurban community. *Clin. Infect. Dis.* **2014**, *58*, 483–491.
7. Lee, N.; Chan, M.C.; Wong, B.; Choi, K.W.; Sin, W.; Lui, G.; Chan, P.K.; Lai, R.W.; Cockram, C.S.; Sung, J.J.; et al. Fecal viral concentration and diarrhea in norovirus gastroenteritis. *Emerg. Infect. Dis.* **2007**, *13*, 1399–1401.
8. van Asten, L.; Siebenga, J.; van den Wijngaard, C.; Verheij, R.; van Vliet, H.; Kretzschmar, M.; Boshuizen, H.; van Pelt, W.; Koopmans, M. Unspecified gastroenteritis illness and deaths in the elderly associated with norovirus epidemics. *Epidemiology* **2011**, *22*, 336–343.
9. Bok, K.; Green, K.Y. Norovirus gastroenteritis in immunocompromised patients. *N. Engl. J. Med.* **2012**, *367*, 2126–2132.
10. Payne, D.C.; Vinje, J.; Szilagyi, P.G.; Edwards, K.M.; Staat, M.A.; Weinberg, G.A.; Hall, C.B.; Chappell, J.; Bernstein, D.I.; Curns, A.T.; et al. Norovirus and medically attended gastroenteritis in U.S. children. *N. Engl. J. Med.* **2013**, *368*, 1121–1130.
11. Hickman, D.; Jones, M.K.; Zhu, S.; Kirkpatrick, E.; Ostrov, D.A.; Wang, X.; Ukhanova, M.; Sun, Y.; Mai, V.; Salemi, M.; et al. The effect of malnutrition on norovirus infection. *MBio* **2014**, *5*, e01032-13.
12. Bartsch, S.M.; Lopman, B.A.; Ozawa, S.; Hall, A.J.; Lee, B.Y. Global Economic Burden of Norovirus Gastroenteritis. *PLoS ONE* **2016**, *11*, e0151219.
13. Scallan, E.; Hoekstra, R.M.; Angulo, F.J.; Tauxe, R.V.; Widdowson, M.A.; Roy, S.L.; Jones, J.L.; Griffin, P.M. Foodborne illness acquired in the United States—Major pathogens. *Emerg. Infect. Dis.* **2011**, *17*, 7–15.

14. Hoffmann, S.; Batz, M.B.; Morris, J.G.; Jr. Annual cost of illness and quality-adjusted life year losses in the United States due to 14 foodborne pathogens. *J. Food Prot.* **2012**, *75*, 1292–1302.
15. Netzler, N.E.; Enosi Tuipulotu, D.; White, P.A. Norovirus antivirals: Where are we now? *Med. Res. Rev.* **2019**, *39*, 860–886, doi:10.1002/med.21545.
16. Hardy, M.E. Norovirus protein structure and function. *FEMS Microbiol. Lett.* **2005**, *253*, 1–8, doi:10.1016/j.femsle.2005.08.031.
17. Prasad, B.V.; Hardy, M.E.; Dokland, T.; Bella, J.; Rossmann, M.G.; Estes, M.K. X-ray crystallographic structure of the Norwalk virus capsid. *Science* **1999**, *286*, 287–290.
18. Shanker, S.; Czako, R.; Sankaran, B.; Atmar, R.L.; Estes, M.K.; Prasad, B.V. Structural analysis of determinants of histo-blood group antigen binding specificity in genogroup I noroviruses. *J. Virol.* **2014**, *88*, 6168–6180.
19. Glass, P.J.; Zeng, C.Q.; Estes, M.K. Two nonoverlapping domains on the Norwalk virus open reading frame 3 (ORF3) protein are involved in the formation of the phosphorylated 35K protein and in ORF3-capsid protein interactions. *J. Virol.* **2003**, *77*, 3569–3577.
20. Smith, T.J. Structural studies on antibody recognition and neutralization of viruses. *Curr. Opin. Virol.* **2011**, *1*, 150–156.
21. Tan, M.; Jiang, X. Norovirus and its histo-blood group antigen receptors: An answer to a historical puzzle. *Trends Microbiol.* **2005**, *13*, 285–293.
22. Singh, B.K.; Leuthold, M.M.; Hansman, G.S. Human noroviruses' fondness for histo-blood group antigens. *J. Virol.* **2015**, *89*, 2024–2040.
23. Zheng, D.P.; Ando, T.; Fankhauser, R.L.; Beard, R.S.; Glass, R.I.; Monroe, S.S. Norovirus classification and proposed strain nomenclature. *Virology* **2006**, *346*, 312–323.
24. Kroneman, A.; Vega, E.; Vennema, H.; Vinje, J.; White, P.A.; Hansman, G.; Green, K.; Martella, V.; Katayama, K.; Koopmans, M. Proposal for a unified norovirus nomenclature and genotyping. *Arch. Virol.* **2013**, *158*, 2059–2068.
25. Vinje, J. Advances in laboratory methods for detection and typing of norovirus. *J. Clin. Microbiol.* **2015**, *53*, 373–381.
26. Schneider, W.L.; Roossinck, M.J. Genetic diversity in RNA virus quasispecies is controlled by host-virus interactions. *J. Virol.* **2001**, *75*, 6566–6571.
27. Lauring, A.S.; Frydman, J.; Andino, R. The role of mutational robustness in RNA virus evolution. *Nat. Rev. Microbiol.* **2013**, *11*, 327–336.
28. Bull, R.A.; Tu, E.T.; McIver, C.J.; Rawlinson, W.D.; White, P.A. Emergence of a new norovirus genotype II.4 variant associated with global outbreaks of gastroenteritis. *J. Clin. Microbiol.* **2006**, *44*, 327–333.
29. Patel, M.M.; Hall, A.J.; Vinje, J.; Parashar, U.D. Noroviruses: A comprehensive review. *J. Clin. Virol.* **2009**, *44*, 1–8.
30. Bok, K.; Abente, E.J.; Realpe-Quintero, M.; Mitra, T.; Sosnovtsev, S.V.; Kapikian, A.Z.; Green, K.Y. Evolutionary dynamics of GII.4 noroviruses over a 34-year period. *J. Virol.* **2009**, *83*, 11890–11901.
31. Zakikhany, K.; Allen, D.J.; Brown, D.; Iturriza-Gomara, M. Molecular evolution of GII-4 Norovirus strains. *PLoS ONE* **2012**, *7*, e41625.
32. Siebenga, J.J.; Vennema, H.; Renckens, B.; de Bruin, E.; van der Veer, B.; Siezen, R.J.; Koopmans, M. Epochal evolution of GGII.4 norovirus capsid proteins from 1995 to 2006. *J. Virol.* **2007**, *81*, 9932–9941.
33. Lindesmith, L.C.; Donaldson, E.F.; Lobue, A.D.; Cannon, J.L.; Zheng, D.P.; Vinje, J.; Baric, R.S. Mechanisms of GII.4 norovirus persistence in human populations. *PLoS Med.* **2008**, *5*, e31.
34. Allen, D.J.; Noad, R.; Samuel, D.; Gray, J.J.; Roy, P.; Iturriza-Gomara, M. Characterisation of a GII-4 norovirus variant-specific surface-exposed site involved in antibody binding. *Virol. J.* **2009**, *6*, 150.
35. Lindesmith, L.C.; Donaldson, E.F.; Baric, R.S. Norovirus GII.4 strain antigenic variation. *J. Virol.* **2011**, *85*, 231–242.
36. Lindesmith, L.C.; Beltramello, M.; Donaldson, E.F.; Corti, D.; Swanstrom, J.; Debbink, K.; Lanzavecchia, A.; Baric, R.S. Immunogenetic mechanisms driving norovirus GII.4 antigenic variation. *PLoS Pathog.* **2012**, *8*, e1002705.
37. Lindesmith, L.C.; Costantini, V.; Swanstrom, J.; Debbink, K.; Donaldson, E.F.; Vinje, J.; Baric, R.S. Emergence of a norovirus GII.4 strain correlates with changes in evolving blockade epitopes. *J. Virol.* **2013**, *87*, 2803–2813.
38. Debbink, K.; Lindesmith, L.C.; Donaldson, E.F.; Costantini, V.; Beltramello, M.; Corti, D.; Swanstrom, J.; Lanzavecchia, A.; Vinje, J.; Baric, R.S. Emergence of new pandemic GII.4 Sydney norovirus strain correlates with escape from herd immunity. *J. Infect. Dis.* **2013**, *208*, 1877–1887.
39. de Graaf, M.; van Beek, J.; Vennema, H.; Podkolzin, A.T.; Hewitt, J.; Bucardo, F.; Templeton, K.; Mans, J.; Nordgren, J.; Reuter, G.; et al. Emergence of a novel GII.17 norovirus—End of the GII.4 era? *Euro Surveill.* **2015**, *20*, 21178.
40. Zhang, X.F.; Huang, Q.; Long, Y.; Jiang, X.; Zhang, T.; Tan, M.; Zhang, Q.L.; Huang, Z.Y.; Li, Y.H.; Ding, Y.Q.; et al. An outbreak caused by GII.17 norovirus with a wide spectrum of HBGA-associated susceptibility. *Sci. Rep.* **2015**, *5*, 17687.
41. Singh, B.K.; Koromyslova, A.; Hefele, L.; Gurth, C.; Hansman, G.S. Structural Evolution of the Emerging 2014-2015 GII.17 Noroviruses. *J. Virol.* **2015**, *90*, 2710–2715.
42. Lu, J.; Fang, L.; Zheng, H.; Lao, J.; Yang, F.; Sun, L.; Xiao, J.; Lin, J.; Song, T.; Ni, T.; et al. The Evolution and Transmission of Epidemic GII.17 Noroviruses. *J. Infect. Dis.* **2016**, *214*, 556–564.
43. Parra, G.I.; Squires, R.B.; Karangwa, C.K.; Johnson, J.A.; Lepore, C.J.; Sosnovtsev, S.V.; Green, K.Y. Static and Evolving Norovirus Genotypes: Implications for Epidemiology and Immunity. *PLoS Pathog.* **2017**, *13*, e1006136.
44. Todd, K.V.; Tripp, R.A. Human Norovirus: Experimental Models of Infection. *Viruses* **2019**, *11*, 151.

45. Chachu, K.A.; Strong, D.W.; LoBue, A.D.; Wobus, C.E.; Baric, R.S.; Virgin, H.W.t. Antibody is critical for the clearance of murine norovirus infection. *J. Virol.* **2008**, *82*, 6610–6617.
46. Grau, K.R.; Roth, A.N.; Zhu, S.; Hernandez, A.; Colliou, N.; DiVita, B.B.; Philip, D.T.; Riffe, C.; Giasson, B.; Wallet, S.M.; et al. The major targets of acute norovirus infection are immune cells in the gut-associated lymphoid tissue. *Nat. Microbiol.* **2017**, *2*, 1586–1591.
47. Gonzalez-Hernandez, M.B.; Liu, T.; Payne, H.C.; Stencel-Baerenwald, J.E.; Ikizler, M.; Yagita, H.; Dermody, T.S.; Williams, I.R.; Wobus, C.E. Efficient norovirus and reovirus replication in the mouse intestine requires microfold (M) cells. *J. Virol.* **2014**, *88*, 6934–6943.
48. Perry, J.W.; Taube, S.; Wobus, C.E. Murine norovirus-1 entry into permissive macrophages and dendritic cells is pH-independent. *Virus Res.* **2009**, *143*, 125–129.
49. Wobus, C.E.; Karst, S.M.; Thackray, L.B.; Chang, K.O.; Sosnovtsev, S.V.; Belliot, G.; Krug, A.; Mackenzie, J.M.; Green, K.Y.; Virgin, H.W. Replication of Norovirus in cell culture reveals a tropism for dendritic cells and macrophages. *PLoS Biol.* **2004**, *2*, e432.
50. Jones, M.K.; Watanabe, M.; Zhu, S.; Graves, C.L.; Keyes, L.R.; Grau, K.R.; Gonzalez-Hernandez, M.B.; Iovine, N.M.; Wobus, C.E.; Vinje, J.; et al. Enteric bacteria promote human and mouse norovirus infection of B cells. *Science* **2014**, *346*, 755–759.
51. Karst, S.M.; Wobus, C.E.; Lay, M.; Davidson, J.; Virgin, H.W.t. STAT1-dependent innate immunity to a Norwalk-like virus. *Science* **2003**, *299*, 1575–1578.
52. Mumphrey, S.M.; Changotra, H.; Moore, T.N.; Heimann-Nichols, E.R.; Wobus, C.E.; Reilly, M.J.; Moghadamfalahi, M.; Shukla, D.; Karst, S.M. Murine norovirus 1 infection is associated with histopathological changes in immunocompetent hosts, but clinical disease is prevented by STAT1-dependent interferon responses. *J. Virol.* **2007**, *81*, 3251–3263.
53. Changotra, H.; Jia, Y.; Moore, T.N.; Liu, G.; Kahan, S.M.; Sosnovtsev, S.V.; Karst, S.M. Type I and type II interferons inhibit the translation of murine norovirus proteins. *J. Virol.* **2009**, *83*, 5683–5692.
54. Chachu, K.A.; LoBue, A.D.; Strong, D.W.; Baric, R.S.; Virgin, H.W. Immune mechanisms responsible for vaccination against and clearance of mucosal and lymphatic norovirus infection. *PLoS Pathog.* **2008**, *4*, e1000236.
55. Waugh, E.; Chen, A.; Baird, M.A.; Brown, C.M.; Ward, V.K. Characterization of the chemokine response of RAW264.7 cells to infection by murine norovirus. *Virus Res.* **2014**, *181*, 27–34.
56. Groom, J.R.; Luster, A.D. CXCR3 ligands: Redundant, collaborative and antagonistic functions. *Immunol. Cell Biol.* **2011**, *89*, 207–215.
57. Park, K.; Cha, K.E.; Myung, H. Observation of inflammatory responses in mice orally fed with bacteriophage T7. *J. Appl. Microbiol.* **2014**, *117*, 627–633.
58. Tomov, V.T.; Osborne, L.C.; Dolfi, D.V.; Sonnenberg, G.F.; Monticelli, L.A.; Mansfield, K.; Virgin, H.W.; Artis, D.; Wherry, E.J. Persistent enteric murine norovirus infection is associated with functionally suboptimal virus-specific CD8 T cell responses. *J. Virol.* **2013**, *87*, 7015–7031.
59. Wilen, C.B.; Lee, S.; Hsieh, L.L.; Orchard, R.C.; Desai, C.; Hykes, B.L., Jr.; McAllaster, M.R.; Balce, D.R.; Feehley, T.; Brestoff, J.R.; et al. Tropism for tuft cells determines immune promotion of norovirus pathogenesis. *Science* **2018**, *360*, 204–208.
60. Tomov, V.T.; Palko, O.; Lau, C.W.; Pattekar, A.; Sun, Y.; Tacheva, R.; Bengsch, B.; Manne, S.; Cosma, G.L.; Eisenlohr, L.C.; et al. Differentiation and Protective Capacity of Virus-Specific CD8(+) T Cells Suggest Murine Norovirus Persistence in an Immune-Privileged Enteric Niche. *Immunity* **2017**, *47*, 723–738.e5.
61. Nice, T.J.; Baldridge, M.T.; McCune, B.T.; Norman, J.M.; Lazear, H.M.; Artyomov, M.; Diamond, M.S.; Virgin, H.W. Interferon-lambda cures persistent murine norovirus infection in the absence of adaptive immunity. *Science* **2015**, *347*, 269–273.
62. Turula, H.; Bragazzi Cunha, J.; Mainou, B.A.; Ramakrishnan, S.K.; Wilke, C.A.; Gonzalez-Hernandez, M.B.; Pry, A.; Fava, J.; Bassis, C.M.; Edelman, J.; et al. Natural Secretory Immunoglobulins Promote Enteric Viral Infections. *J. Virol.* **2018**, *92*, e00826-18.
63. Zhu, S.; Regev, D.; Watanabe, M.; Hickman, D.; Moussatche, N.; Jesus, D.M.; Kahan, S.M.; Napthine, S.; Brierley, I.; Hunter, R.N., 3rd; et al. Identification of immune and viral correlates of norovirus protective immunity through comparative study of intra-cluster norovirus strains. *PLoS Pathog.* **2013**, *9*, e1003592.
64. Mora, J.R. Homing imprinting and immunomodulation in the gut: Role of dendritic cells and retinoids. *Inflamm. Bowel Dis.* **2008**, *14*, 275–289.
65. Kolawole, A.O.; Xia, C.; Li, M.; Gamez, M.; Yu, C.; Rippinger, C.M.; Yucha, R.E.; Smith, T.J.; Wobus, C.E. Newly isolated mAbs broaden the neutralizing epitope in murine norovirus. *J. Gen. Virol.* **2014**, *95*, 1958–1968.
66. Wobus, C.E.; Thackray, L.B.; Virgin, H.W. Murine Norovirus: A Model System To Study Norovirus Biology and Pathogenesis. *J. Virol.* **2006**, *80*, 5104–5112.
67. Thackray, L.B.; Wobus, C.E.; Chachu, K.A.; Liu, B.; Alegre, E.R.; Henderson, K.S.; Kelley, S.T.; Virgin, H.W., 4th. Murine noroviruses comprising a single genogroup exhibit biological diversity despite limited sequence divergence. *J. Virol.* **2007**, *81*, 10460–10473.
68. Ettayebi, K.; Crawford, S.E.; Murakami, K.; Broughman, J.R.; Karandikar, U.; Tenge, V.R.; Neill, F.H.; Blutt, S.E.; Zeng, X.-L.; Qu, L.; et al. Replication of human noroviruses in stem cell–derived human enteroids. **2016**, *353*, 1387–1393, doi:10.1126/science.aaf5211.
69. Karandikar, U.C.; Crawford, S.E.; Ajami, N.J.; Murakami, K.; Kou, B.; Ettayebi, K.; Papanicolaou, G.A.; Jongwutiwes, U.; Perales, M.A.; Shia, J.; et al. Detection of human norovirus in intestinal biopsies from immunocompromised transplant patients. *J. Gen. Virol.* **2016**, *97*, 2291–2300.
70. Taube, S.; Perry, J.W.; Yetming, K.; Patel, S.P.; Auble, H.; Shu, L.; Nawar, H.F.; Lee, C.H.; Connell, T.D.; Shayman, J.A.; et al. Ganglioside-linked terminal sialic acid moieties on murine macrophages function as attachment receptors for murine noroviruses. *J. Virol.* **2009**, *83*, 4092–4101.

71. Hutson, A.M.; Atmar, R.L.; Marcus, D.M.; Estes, M.K. Norwalk virus-like particle hemagglutination by binding to h histo-blood group antigens. *J. Virol.* **2003**, *77*, 405–415.
72. Orchard, R.C.; Wilen, C.B.; Doench, J.G.; Baldridge, M.T.; McCune, B.T.; Lee, Y.C.; Lee, S.; Pruett-Miller, S.M.; Nelson, C.A.; Fremont, D.H.; et al. Discovery of a proteinaceous cellular receptor for a norovirus. *Science* **2016**, *353*, 933–936.
73. Newman, K.L.; Moe, C.L.; Kirby, A.E.; Flanders, W.D.; Parkos, C.A.; Leon, J.S. Human norovirus infection and the acute serum cytokine response. *Clin. Exp. Immunol.* **2015**, *182*, 195–203.
74. Schreiber, D.S.; Blacklow, N.R.; Trier, J.S. The mucosal lesion of the proximal small intestine in acute infectious nonbacterial gastroenteritis. *N. Engl. J. Med.* **1973**, *288*, 1318–1323.
75. Long, K.Z.; Garcia, C.; Ko, G.; Santos, J.I.; Al Mamun, A.; Rosado, J.L.; DuPont, H.L.; Nathakumar, N. Vitamin A modifies the intestinal chemokine and cytokine responses to norovirus infection in Mexican children. *J. Nutr.* **2011**, *141*, 957–963.
76. Lindesmith, L.; Moe, C.; Lependu, J.; Frelinger, J.A.; Treanor, J.; Baric, R.S. Cellular and humoral immunity following Snow Mountain virus challenge. *J. Virol.* **2005**, *79*, 2900–2909.
77. Ponterio, E.; Petrizzo, A.; Di Bartolo, I.; Buonaguro, F.M.; Buonaguro, L.; Ruggeri, F.M. Pattern of activation of human antigen presenting cells by genotype GII.4 norovirus virus-like particles. *J. Transl. Med.* **2013**, *11*, 127.
78. Lindesmith, L.C.; Donaldson, E.; Leon, J.; Moe, C.L.; Frelinger, J.A.; Johnston, R.E.; Weber, D.J.; Baric, R.S. Heterotypic humoral and cellular immune responses following Norwalk virus infection. *J. Virol.* **2010**, *84*, 1800–1815.
79. Chen, S.M.; Lin, C.P.; Tsai, J.D.; Chao, Y.H.; Sheu, J.N. The significance of serum and fecal levels of interleukin-6 and interleukin-8 in hospitalized children with acute rotavirus and norovirus gastroenteritis. *Pediatr. Neonatol.* **2014**, *55*, 120–126.
80. Malm, M.; Hyoty, H.; Knip, M.; Vesikari, T.; Blazevic, V. Development of T cell immunity to norovirus and rotavirus in children under five years of age. *Sci. Rep.* **2019**, *9*, 3199.
81. Malm, M.; Tamminen, K.; Vesikari, T.; Blazevic, V. Norovirus-Specific Memory T Cell Responses in Adult Human Donors. *Front. Microbiol.* **2016**, *7*, 1570.
82. Tacket, C.O.; Sztein, M.B.; Losonsky, G.A.; Wasserman, S.S.; Estes, M.K. Humoral, mucosal, and cellular immune responses to oral Norwalk virus-like particles in volunteers. *Clin. Immunol.* **2003**, *108*, 241–247.
83. Koo, H.L.; Ajami, N.; Atmar, R.L.; DuPont, H.L. Noroviruses: The leading cause of gastroenteritis worldwide. *Discov. Med.* **2010**, *10*, 61–70.
84. Johnson, P.C.; Mathewson, J.J.; DuPont, H.L.; Greenberg, H.B. Multiple-challenge study of host susceptibility to Norwalk gastroenteritis in US adults. *J. Infect. Dis.* **1990**, *161*, 18–21.
85. Okhuysen, P.C.; Jiang, X.; Ye, L.; Johnson, P.C.; Estes, M.K. Viral shedding and fecal IgA response after Norwalk virus infection. *J. Infect. Dis.* **1995**, *171*, 566–569.
86. Lindesmith, L.C.; Ferris, M.T.; Mullan, C.W.; Ferreira, J.; Debbink, K.; Swanstrom, J.; Richardson, C.; Goodwin, R.R.; Baehner, F.; Mendelman, P.M.; et al. Broad Blockade Antibody Responses in Human Volunteers after Immunization with a Multivalent Norovirus VLP Candidate Vaccine: Immunological Analyses from a Phase I Clinical Trial. *PLoS Med.* **2015**, *12*, e1001807.
87. Bok, K.; Parra, G.I.; Mitra, T.; Abente, E.; Shaver, C.K.; Boon, D.; Engle, R.; Yu, C.; Kapikian, A.Z.; Sosnovtsev, S.V.; et al. Chimpanzees as an animal model for human norovirus infection and vaccine development. *Proc. Natl. Acad. Sci. USA* **2011**, *108*, 325–330.
88. Chen, Z.; Sosnovtsev, S.V.; Bok, K.; Parra, G.I.; Makiya, M.; Agulto, L.; Green, K.Y.; Purcell, R.H. Development of Norwalk virus-specific monoclonal antibodies with therapeutic potential for the treatment of Norwalk virus gastroenteritis. *J. Virol.* **2013**, *87*, 9547–9557.
89. Woodward, J.; Gkrania-Klotsas, E.; Kumararatne, D. Chronic norovirus infection and common variable immunodeficiency. *Clin. Exp. Immunol*. **2017**, *188*, 363–370.
90. Knoll, B.M.; Lindesmith, L.C.; Yount, B.L.; Baric, R.S.; Marty, F.M. Resolution of diarrhea in an immunocompromised patient with chronic norovirus gastroenteritis correlates with constitution of specific antibody blockade titer. *Infection* **2016**, *44*, 551–554.
91. Green, K.Y.; Lew, J.F.; Jiang, X.; Kapikian, A.Z.; Estes, M.K. Comparison of the reactivities of baculovirus-expressed recombinant Norwalk virus capsid antigen with those of the native Norwalk virus antigen in serologic assays and some epidemiologic observations. *J. Clin. Microbiol.* **1993**, *31*, 2185–2191.
92. White, L.J.; Ball, J.M.; Hardy, M.E.; Tanaka, T.N.; Kitamoto, N.; Estes, M.K. Attachment and entry of recombinant Norwalk virus capsids to cultured human and animal cell lines. *J. Virol.* **1996**, *70*, 6589–6597.
93. Jiang, X.; Wang, M.; Graham, D.Y.; Estes, M.K. Expression, self-assembly, and antigenicity of the Norwalk virus capsid protein. *J. Virol.* **1992**, *66*, 6527–6532.
94. Harrington, P.R.; Yount, B.; Johnston, R.E.; Davis, N.; Moe, C.; Baric, R.S. Systemic, mucosal, and heterotypic immune induction in mice inoculated with Venezuelan equine encephalitis replicons expressing Norwalk virus-like particles. *J. Virol.* **2002**, *76*, 730–742.
95. Taube, S.; Kurth, A.; Schreier, E. Generation of recombinant Norovirus-like particles (VLP) in the human endothelial kidney cell line 293T. *Arch. Virol.* **2005**, *150*, 1425–1431.
96. Zhang, X.; Buehner, N.A.; Hutson, A.M.; Estes, M.K.; Mason, H.S. Tomato is a highly effective vehicle for expression and oral immunization with Norwalk virus capsid protein. *Plant Biotechnol. J.* **2006**, *4*, 419–432.
97. Santi, L.; Batchelor, L.; Huang, Z.; Hjelm, B.; Kilbourne, J.; Arntzen, C.J.; Chen, Q.; Mason, H.S. An efficient plant viral expression system generating orally immunogenic Norwalk virus-like particles. *Vaccine* **2008**, *26*, 1846–1854.
98. Lindesmith, L.C.; Donaldson, E.F.; Beltramello, M.; Pintus, S.; Corti, D.; Swanstrom, J.; Debbink, K.; Jones, T.A.; Lanzavecchia, A.; Baric, R.S. Particle conformation regulates antibody access to a conserved GII.4 norovirus blockade epitope. *J. Virol.* **2014**, *88*, 8826–8842.

99. Tian, P.; Yang, D.; Jiang, X.; Zhong, W.; Cannon, J.L.; Burkhardt, W., 3rd; Woods, J.W.; Hartman, G.; Lindesmith, L.; Baric, R.S.; et al. Specificity and kinetics of norovirus binding to magnetic bead-conjugated histo-blood group antigens. *J. Appl. Microbiol.* **2010**, *109*, 1753–1762.
100. Kou, B.; Crawford, S.E.; Ajami, N.J.; Czako, R.; Neill, F.H.; Tanaka, T.N.; Kitamoto, N.; Palzkill, T.G.; Estes, M.K.; Atmar, R.L. Characterization of cross-reactive norovirus-specific monoclonal antibodies. *Clin. Vaccine Immunol.* **2015**, *22*, 160–167.
101. Rydell, G.E.; Nilsson, J.; Rodriguez-Diaz, J.; Ruvoen-Clouet, N.; Svensson, L.; Le Pendu, J.; Larson, G. Human noroviruses recognize sialyl Lewis x neoglycoprotein. *Glycobiology* **2009**, *19*, 309–320.
102. Garaicoechea, L.; Aguilar, A.; Parra, G.I.; Bok, M.; Sosnovtsev, S.V.; Canziani, G.; Green, K.Y.; Bok, K.; Parreño, V. Llama Nanoantibodies with Therapeutic Potential against Human Norovirus Diarrhea. *PLoS ONE* **2015**, *10*, e0133665.
103. Debbink, K.; Donaldson, E.F.; Lindesmith, L.C.; Baric, R.S. Genetic mapping of a highly variable norovirus GII.4 blockade epitope: Potential role in escape from human herd immunity. *J. Virol.* **2012**, *86*, 1214–1226.
104. Parra, G.I.; Abente, E.J.; Sandoval-Jaime, C.; Sosnovtsev, S.V.; Bok, K.; Green, K.Y. Multiple antigenic sites are involved in blocking the interaction of GII.4 norovirus capsid with ABH histo-blood group antigens. *J. Virol.* **2012**, *86*, 7414–7426.
105. Debbink, K.; Lindesmith, L.C.; Donaldson, E.F.; Swanstrom, J.; Baric, R.S. Chimeric GII.4 norovirus virus-like-particle-based vaccines induce broadly blocking immune responses. *J. Virol.* **2014**, *88*, 7256–7266.
106. Parker, T.D.; Kitamoto, N.; Tanaka, T.; Hutson, A.M.; Estes, M.K. Identification of Genogroup I and Genogroup II broadly reactive epitopes on the norovirus capsid. *J. Virol.* **2005**, *79*, 7402–7409.
107. Swanstrom, J.; Lindesmith, L.C.; Donaldson, E.F.; Yount, B.; Baric, R.S. Characterization of blockade antibody responses in GII.2.1976 Snow Mountain virus-infected subjects. *J. Virol.* **2014**, *88*, 829–837.
108. Tan, M.; Hegde, R.S.; Jiang, X. The P domain of norovirus capsid protein forms dimer and binds to histo-blood group antigen receptors. *J. Virol.* **2004**, *78*, 6233–6242.
109. Tan, M.; Fang, P.; Chachiyo, T.; Xia, M.; Huang, P.; Fang, Z.; Jiang, W.; Jiang, X. Noroviral P particle: Structure, function and applications in virus-host interaction. *Virology* **2008**, *382*, 115–123.
110. Tan, M.; Fang, P.A.; Xia, M.; Chachiyo, T.; Jiang, W.; Jiang, X. Terminal modifications of norovirus P domain resulted in a new type of subviral particles, the small P particles. *Virology* **2011**, *410*, 345–352.
111. Tan, M.; Jiang, X. The p domain of norovirus capsid protein forms a subviral particle that binds to histo-blood group antigen receptors. *J. Virol.* **2005**, *79*, 14017–14030.
112. Tamminen, K.; Huhti, L.; Koho, T.; Lappalainen, S.; Hytonen, V.P.; Vesikari, T.; Blazevic, V. A comparison of immunogenicity of norovirus GII-4 virus-like particles and P-particles. *Immunology* **2012**, *135*, 89–99.
113. Fang, H.; Tan, M.; Xia, M.; Wang, L.; Jiang, X. Norovirus P particle efficiently elicits innate, humoral and cellular immunity. *PLoS ONE* **2013**, *8*, e63269.
114. Batten, C.A.; Clarke, I.N.; Kempster, S.L.; Oliver, S.L.; Bridger, J.C.; Lambden, P.R. Characterization of a cross-reactive linear epitope in human genogroup I and bovine genogroup III norovirus capsid proteins. *Virology* **2006**, *356*, 179–187.
115. Oliver, S.L.; Batten, C.A.; Deng, Y.; Elschner, M.; Otto, P.; Charpilienne, A.; Clarke, I.N.; Bridger, J.C.; Lambden, P.R. Genotype 1 and genotype 2 bovine noroviruses are antigenically distinct but share a cross-reactive epitope with human noroviruses. *J. Clin. Microbiol.* **2006**, *44*, 992–998.
116. Li, X.; Zhou, R.; Tian, X.; Li, H.; Zhou, Z. Characterization of a cross-reactive monoclonal antibody against Norovirus genogroups I, II, III and V. *Virus Res.* **2010**, *151*, 142–147.
117. Gray, J.J.; Cunliffe, C.; Ball, J.; Graham, D.Y.; Desselberger, U.; Estes, M.K. Detection of immunoglobulin M (IgM), IgA, and IgG Norwalk virus-specific antibodies by indirect enzyme-linked immunosorbent assay with baculovirus-expressed Norwalk virus capsid antigen in adult volunteers challenged with Norwalk virus. *J. Clin. Microbiol.* **1994**, *32*, 3059–3063.
118. Alvarado, G.; Ettayebi, K.; Atmar, R.L.; Bombardi, R.G.; Kose, N.; Estes, M.K.; Crowe, J.E., Jr. Human Monoclonal Antibodies That Neutralize Pandemic GII.4 Noroviruses. *Gastroenterology* **2018**, *155*, 1898–1907.
119. Koromyslova, A.D.; Morozov, V.A.; Hefele, L.; Hansman, G.S. Human Norovirus Neutralized by a Monoclonal Antibody Targeting the HBGA Pocket. *J. Virol.* **2019**, *93*, e02174-18, doi:10.1128/JVI.02174-18.
120. Tamminen, K.; Malm, M.; Vesikari, T.; Blazevic, V. Norovirus-specific mucosal antibodies correlate to systemic antibodies and block norovirus virus-like particles binding to histo-blood group antigens. *Clin. Immunol.* **2018**, *197*, 110–117.
121. Lindesmith, L.C.; Beltramello, M.; Swanstrom, J.; Jones, T.A.; Corti, D.; Lanzavecchia, A.; Baric, R.S. Serum Immunoglobulin A Cross-Strain Blockade of Human Noroviruses. *Open Forum Infect. Dis.* **2015**, *2*, ofv084.
122. Lindesmith, L.; Moe, C.; Marionneau, S.; Ruvoen, N.; Jiang, X.; Lindblad, L.; Stewart, P.; LePendu, J.; Baric, R. Human susceptibility and resistance to Norwalk virus infection. *Nat. Med.* **2003**, *9*, 548–553.

123. Ramani, S.; Neill, F.H.; Opekun, A.R.; Gilger, M.A.; Graham, D.Y.; Estes, M.K.; Atmar, R.L. Mucosal and Cellular Immune Responses to Norwalk Virus. *J. Infect. Dis.* **2015**, *212*, 397–405.
124. Sapparapu, G.; Czako, R.; Alvarado, G.; Shanker, S.; Prasad, B.V.; Atmar, R.L.; Estes, M.K.; Crowe, J.E., Jr. Frequent Use of the IgA Isotype in Human B Cells Encoding Potent Norovirus-Specific Monoclonal Antibodies That Block HBGA Binding. *PLoS Pathog.* **2016**, *12*, e1005719.
125. Shanker, S.; Czako, R.; Sapparapu, G.; Alvarado, G.; Viskovska, M.; Sankaran, B.; Atmar, R.L.; Crowe, J.E., Jr.; Estes, M.K.; Prasad, B.V. Structural basis for norovirus neutralization by an HBGA blocking human IgA antibody. *Proc. Natl. Acad. Sci. USA* **2016**, *113*, E5830–E5837.
126. Muyldermans, S. Nanobodies: Natural single-domain antibodies. *Annu. Rev. Biochem.* **2013**, *82*, 775–797.
127. Koromyslova, A.D.; Hansman, G.S. Nanobody binding to a conserved epitope promotes norovirus particle disassembly. *J. Virol.* **2015**, *89*, 2718–2730.
128. Doerflinger, S.Y.; Tabatabai, J.; Schnitzler, P.; Farah, C.; Rameil, S.; Sander, P.; Koromyslova, A.; Hansman, G.S. Development of a Nanobody-Based Lateral Flow Immunoassay for Detection of Human Norovirus. *mSphere* **2016**, *1*, e00219-16.
129. Koromyslova, A.D.; Hansman, G.S. Nanobodies targeting norovirus capsid reveal functional epitopes and potential mechanisms of neutralization. *PLoS Pathog.* **2017**, *13*, e1006636.
130. Ruoff, K.; Kilic, T.; Devant, J.; Koromyslova, A.; Ringel, A.; Hempelmann, A.; Geiss, C.; Graf, J.; Haas, M.; Roggenbach, I.; et al. Structural Basis of Nanobodies Targeting the Prototype Norovirus. *J. Virol.* **2019**, *93*, e02005-18.
131. Huston, J.S.; McCartney, J.; Tai, M.S.; Mottola-Hartshorn, C.; Jin, D.; Warren, F.; Keck, P.; Oppermann, H. Medical applications of single-chain antibodies. *Int. Rev. Immunol.* **1993**, *10*, 195–217.
132. Parra, G.I.; Sosnovtsev, S.V.; Abente, E.J.; Sandoval-Jaime, C.; Bok, K.; Dolan, M.A.; Green, K.Y. Mapping and modeling of a strain-specific epitope in the Norwalk virus capsid inner shell. *Virology* **2016**, *492*, 232–241.
133. Burton-MacLeod, J.A.; Kane, E.M.; Beard, R.S.; Hadley, L.A.; Glass, R.I.; Ando, T. Evaluation and comparison of two commercial enzyme-linked immunosorbent assay kits for detection of antigenically diverse human noroviruses in stool samples. *J. Clin. Microbiol.* **2004**, *42*, 2587–2595.
134. Kou, B.; Huang, W.; Neill, F.H.; Palzkill, T.; Estes, M.K.; Atmar, R.L. Norovirus Antigen Detection with a Combination of Monoclonal and Single-Chain Antibodies. *J. Clin. Microbiol.* **2015**, *53*, 3916–3918.
135. de Rougemont, A.; Ruvoen-Clouet, N.; Simon, B.; Estienney, M.; Elie-Caille, C.; Aho, S.; Pothier, P.; Le Pendu, J.; Boireau, W.; Belliot, G. Qualitative and Quantitative Analysis of the Binding of GII.4 Norovirus Variants onto Human Blood Group Antigens. *J. Virol.* **2011**, *85*, 4057–4070.
136. Heggelund, J.E.; Varrot, A.; Imberty, A.; Krengel, U. Histo-blood group antigens as mediators of infections. *Curr. Opin. Struct. Biol.* **2017**, *44*, 190–200.
137. Koromyslova, A.D.; Leuthold, M.M.; Bowler, M.W.; Hansman, G.S. The sweet quartet: Binding of fucose to the norovirus capsid. *Virology* **2015**, *483*, 203–208.
138. Le Pendu, J.; Ruvoen-Clouet, N.; Kindberg, E.; Svensson, L. Mendelian resistance to human norovirus infections. *Semin. Immunol.* **2006**, *18*, 375–386.
139. Reeck, A.; Kavanagh, O.; Estes, M.K.; Opekun, A.R.; Gilger, M.A.; Graham, D.Y.; Atmar, R.L. Serological correlate of protection against norovirus-induced gastroenteritis. *J. Infect. Dis.* **2010**, *202*, 1212–1218.
140. Tan, M.; Jiang, X. Norovirus-host interaction: Multi-selections by human histo-blood group antigens. *Trends Microbiol.* **2011**, *19*, 382–388.
141. Shanker, S.; Choi, J.M.; Sankaran, B.; Atmar, R.L.; Estes, M.K.; Prasad, B.V. Structural analysis of histo-blood group antigen binding specificity in a norovirus GII.4 epidemic variant: Implications for epochal evolution. *J. Virol.* **2011**, *85*, 8635–8645.
142. Nordgren, J.; Kindberg, E.; Lindgren, P.E.; Matussek, A.; Svensson, L. Norovirus gastroenteritis outbreak with a secretor-independent susceptibility pattern, Sweden. *Emerg. Infect. Dis.* **2010**, *16*, 81–87.
143. Donaldson, E.F.; Lindesmith, L.C.; Lobue, A.D.; Baric, R.S. Viral shape-shifting: Norovirus evasion of the human immune system. *Nat. Rev. Microbiol.* **2010**, *8*, 231–241.
144. Frenck, R.; Bernstein, D.I.; Xia, M.; Huang, P.; Zhong, W.; Parker, S.; Dickey, M.; McNeal, M.; Jiang, X. Predicting susceptibility to norovirus GII.4 by use of a challenge model involving humans. *J. Infect. Dis.* **2012**, *206*, 1386–1393.
145. Czako, R.; Atmar, R.L.; Opekun, A.R.; Gilger, M.A.; Graham, D.Y.; Estes, M.K. Serum hemagglutination inhibition activity correlates with protection from gastroenteritis in persons infected with Norwalk virus. *Clin. Vaccine Immunol.* **2012**, *19*, 284–287.
146. Lindesmith, L.C.; Debbink, K.; Swanstrom, J.; Vinje, J.; Costantini, V.; Baric, R.S.; Donaldson, E.F. Monoclonal antibody-based antigenic mapping of norovirus GII.4-2002. *J. Virol.* **2012**, *86*, 873–883.
147. Atmar, R.L.; Bernstein, D.I.; Harro, C.D.; Al-Ibrahim, M.S.; Chen, W.H.; Ferreira, J.; Estes, M.K.; Graham, D.Y.; Opekun, A.R.; Richardson, C.; et al. Norovirus vaccine against experimental human Norwalk Virus illness. *N. Engl. J. Med.* **2011**, *365*, 2178–2187.
148. Green, K.Y.; Belliot, G.; Taylor, J.L.; Valdesuso, J.; Lew, J.F.; Kapikian, A.Z.; Lin, F.Y. A predominant role for Norwalk-like viruses as agents of epidemic gastroenteritis in Maryland nursing homes for the elderly. *J. Infect. Dis.* **2002**, *185*, 133–146.

149. Carmona-Vicente, N.; Allen, D.J.; Rodríguez-Díaz, J.; Iturriza-Gómara, M.; Buesa, J. Antibodies against Lewis antigens inhibit the binding of human norovirus GII.4 virus-like particles to saliva but not to intestinal Caco-2 cells. *Virol. J.* **2016**, *13*, 82.
150. Debbink, K.; Lindesmith, L.C.; Ferris, M.T.; Swanstrom, J.; Beltramello, M.; Corti, D.; Lanzavecchia, A.; Baric, R.S. Within-host evolution results in antigenically distinct GII.4 noroviruses. *J. Virol.* **2014**, *88*, 7244–7255.
151. Karst, S.M.; Baric, R.S. What is the reservoir of emergent human norovirus strains? *J. Virol.* **2015**, *89*, 5756–5759.
152. van Beek, J.; de Graaf, M.; Smits, S.; Schapendonk, C.M.E.; Verjans, G.; Vennema, H.; van der Eijk, A.A.; Phan, M.V.T.; Cotten, M.; Koopmans, M. Whole-Genome Next-Generation Sequencing to Study Within-Host Evolution of Norovirus (NoV) Among Immunocompromised Patients With Chronic NoV Infection. *J. Infect. Dis.* **2017**, *216*, 1513–1524.
153. Schorn, R.; Hohne, M.; Meerbach, A.; Bossart, W.; Wuthrich, R.P.; Schreier, E.; Muller, N.J.; Fehr, T. Chronic norovirus infection after kidney transplantation: Molecular evidence for immune-driven viral evolution. *Clin. Infect. Dis.* **2010**, *51*, 307–314.
154. Lindesmith, L.C.; Brewer-Jensen, P.D.; Mallory, M.L.; Yount, B.; Collins, M.H.; Debbink, K.; Graham, R.L.; Baric, R.S. Human Norovirus Epitope D Plasticity Allows Escape from Antibody Immunity without Loss of Capacity for Binding Cellular Ligands. *J. Virol.* **2019**, *93*, e01813-18.
155. Choi, J.M.; Hutson, A.M.; Estes, M.K.; Prasad, B.V. Atomic resolution structural characterization of recognition of histo-blood group antigens by Norwalk virus. *Proc. Natl. Acad. Sci. USA* **2008**, *105*, 9175–9180.
156. Mallagaray, A.; Lockhauserbaumer, J.; Hansman, G.; Uetrecht, C.; Peters, T. Attachment of norovirus to histo blood group antigens: A cooperative multistep process. *Angew. Chem. Int. Ed. Engl.* **2015**, *54*, 12014–12019.
157. Tan, M.; Xia, M.; Chen, Y.; Bu, W.; Hegde, R.S.; Meller, J.; Li, X.; Jiang, X. Conservation of carbohydrate binding interfaces: Evidence of human HBGA selection in norovirus evolution. *PLoS ONE* **2009**, *4*, e5058.
158. Allen, D.J.; Gray, J.J.; Gallimore, C.I.; Xerry, J.; Iturriza-Gomara, M. Analysis of amino acid variation in the P2 domain of the GII-4 norovirus VP1 protein reveals putative variant-specific epitopes. *PLoS ONE* **2008**, *3*, e1485.
159. Siebenga, J.J.; Lemey, P.; Kosakovsky Pond, S.L.; Rambaut, A.; Vennema, H.; Koopmans, M. Phylodynamic reconstruction reveals norovirus GII.4 epidemic expansions and their molecular determinants. *PLoS Pathog.* **2010**, *6*, e1000884.
160. Lindesmith, L.C.; Mallory, M.L.; Debbink, K.; Donaldson, E.F.; Brewer-Jensen, P.D.; Swann, E.W.; Sheahan, T.P.; Graham, R.L.; Beltramello, M.; Corti, D.; et al. Conformational Occlusion of Blockade Antibody Epitopes, a Novel Mechanism of GII.4 Human Norovirus Immune Evasion. *mSphere* **2018**, *3*, e00518-17.
161. Kolawole, A.O.; Smith, H.Q.; Svoboda, S.A.; Lewis, M.S.; Sherman, M.B.; Lynch, G.C.; Pettitt, B.M.; Smith, T.J.; Wobus, C.E. Norovirus Escape from Broadly Neutralizing Antibodies Is Limited to Allostery-Like Mechanisms. *mSphere* **2017**, *2*, e00334-17.
162. Kolawole, A.O.; Li, M.; Xia, C.; Fischer, A.E.; Giacobbi, N.S.; Rippinger, C.M.; Proescher, J.B.; Wu, S.K.; Bessling, S.L.; Gamez, M.; et al. Flexibility in surface-exposed loops in a virus capsid mediates escape from antibody neutralization. *J. Virol.* **2014**, *88*, 4543–4557.
163. Lochridge, V.P.; Jutila, K.L.; Graff, J.W.; Hardy, M.E. Epitopes in the P2 domain of norovirus VP1 recognized by monoclonal antibodies that block cell interactions. *J. Gen. Virol.* **2005**, *86*, 2799–2806.
164. Ettayebi, K.; Hardy, M.E. Recombinant norovirus-specific scFv inhibit virus-like particle binding to cellular ligands. *Virol. J.* **2008**, *5*, 21.
165. Yildiz, M.; Kocak, A. Molecular Dynamics Studies of Histo-Blood Group Antigen Blocking Human Immunoglobulin A Antibody and Escape Mechanism in Noroviruses Upon Mutation. *J. Comput. Biol.* **2018**, doi:10.1089/cmb.2018.0163.
166. Debbink, K.; Lindesmith, L.C.; Donaldson, E.F.; Baric, R.S. Norovirus immunity and the great escape. *PLoS Pathog.* **2012**, *8*, e1002921.
167. Lindesmith, L.C.; Kocher, J.F.; Donaldson, E.F.; Debbink, K.; Mallory, M.L.; Swann, E.W.; Brewer-Jensen, P.D.; Baric, R.S. Emergence of Novel Human Norovirus GII.17 Strains Correlates With Changes in Blockade Antibody Epitopes. *J. Infect. Dis.* **2017**, *216*, 1227–1234.
168. Lindesmith, L.C.; Brewer-Jensen, P.D.; Mallory, M.L.; Debbink, K.; Swann, E.W.; Vinje, J.; Baric, R.S. Antigenic characterization of a novel recombinant GII.P16-GII.4 Sydney norovirus strain with minor sequence variation leading to antibody escape. *J. Infect. Dis.* **2018**, *217*, 1145–1152, doi:10.1093/infdis/jix651.
169. Carmona-Vicente, N.; Vila-Vicent, S.; Allen, D.; Gozalbo-Rovira, R.; Iturriza-Gómara, M.; Buesa, J.; Rodríguez-Díaz, J.; López, S. Characterization of a Novel Conformational GII.4 Norovirus Epitope: Implications for Norovirus-Host Interactions. *J. Virol.* **2016**, *90*, 7703–7714, doi:10.1128/JVI.01023-16.
170. Cannon, J.P.; O'Driscoll, M.; Litman, G.W. Specific lipid recognition is a general feature of CD300 and TREM molecules. *Immunogenetics* **2012**, *64*, 39–47.
171. Kilic, T.; Koromyslova, A.; Malak, V.; Hansman, G.S. Atomic Structure of the Murine Norovirus Protruding Domain and Soluble CD300lf Receptor Complex. *J. Virol.* **2018**, *92*, e00413-18.
172. Nelson, C.A.; Wilen, C.B.; Dai, Y.N.; Orchard, R.C.; Kim, A.S.; Stegeman, R.A.; Hsieh, L.L.; Smith, T.J.; Virgin, H.W.; Fremont, D.H. Structural basis for murine norovirus engagement of bile acids and the CD300lf receptor. *Proc. Natl. Acad. Sci. USA* **2018**, *115*, E9201–E9210.
173. Kilic, T.; Koromyslova, A.; Hansman, G.S. Structural Basis for Human Norovirus Capsid Binding to Bile Acids. *J. Virol.* **2019**, *93*, e01581-18.

174. Taube, S.; Rubin, J.R.; Katpally, U.; Smith, T.J.; Kendall, A.; Stuckey, J.A.; Wobus, C.E. High-resolution x-ray structure and functional analysis of the murine norovirus 1 capsid protein protruding domain. *J. Virol.* **2010**, *84*, 5695–5705.
175. Lochridge, V.P.; Hardy, M.E. A single-amino-acid substitution in the P2 domain of VP1 of murine norovirus is sufficient for escape from antibody neutralization. *J. Virol.* **2007**, *81*, 12316–12322.
176. Huang, W.; Samanta, M.; Crawford, S.E.; Estes, M.K.; Neill, F.H.; Atmar, R.L.; Palzkill, T. Identification of human single-chain antibodies with broad reactivity for noroviruses. *Protein Eng. Des. Sel.* **2014**, *27*, 339–349.
177. Malm, M.; Tamminen, K.; Vesikari, T.; Blazevic, V. Type-specific and cross-reactive antibodies and T cell responses in norovirus VLP immunized mice are targeted both to conserved and variable domains of capsid VP1 protein. *Mol. Immunol.* **2016**, *78*, 27–37.
178. Malm, M.; Uusi-Kerttula, H.; Vesikari, T.; Blazevic, V. High serum levels of norovirus genotype-specific blocking antibodies correlate with protection from infection in children. *J. Infect. Dis.* **2014**, *210*, 1755–1762.
179. Blazevic, V.; Malm, M.; Vesikari, T. Induction of homologous and cross-reactive GII.4-specific blocking antibodies in children after GII.4 New Orleans norovirus infection. *J. Med. Virol.* **2015**, *87*, 1656–1661.
180. Czakó, R.; Atmar, R.L.; Opekun, A.R.; Gilger, M.A.; Graham, D.Y.; Estes, M.K. Experimental Human Infection with Norwalk Virus Elicits a Surrogate Neutralizing Antibody Response with Cross-Genogroup Activity. *Clin. Vaccine Immunol.* **2015**, *22*, 221–228.
181. LoBue, A.D.; Lindesmith, L.; Yount, B.; Harrington, P.R.; Thompson, J.M.; Johnston, R.E.; Moe, C.L.; Baric, R.S. Multivalent norovirus vaccines induce strong mucosal and systemic blocking antibodies against multiple strains. *Vaccine* **2006**, *24*, 5220–5234.
182. Rockx, B.; Baric, R.S.; de Grijs, I.; Duizer, E.; Koopmans, M.P. Characterization of the homo- and heterotypic immune responses after natural norovirus infection. *J. Med. Virol.* **2005**, *77*, 439–446.
183. Strong, D.W.; Thackray, L.B.; Smith, T.J.; Virgin, H.W. Protruding domain of capsid protein is necessary and sufficient to determine murine norovirus replication and pathogenesis in vivo. *J. Virol.* **2012**, *86*, 2950–2958.
184. Kocher, J.; Bui, T.; Giri-Rachman, E.; Wen, K.; Li, G.; Yang, X.; Liu, F.; Tan, M.; Xia, M.; Zhong, W.; et al. Intranasal P Particle Vaccine Provided Partial Cross-Variant Protection against Human GII.4 Norovirus Diarrhea in Gnotobiotic Pigs. *J. Virol.* **2014**, *88*, 9728–9743, doi:10.1128/JVI.01249-14.
185. LoBue, A.D.; Lindesmith, L.C.; Baric, R.S. Identification of cross-reactive norovirus CD4+ T cell epitopes. *J. Virol.* **2010**, *84*, 8530–8538.
186. Ellis, J.M.; Henson, V.; Slack, R.; Ng, J.; Hartzman, R.J.; Katovich Hurley, C. Frequencies of HLA-A2 alleles in five U.S. population groups. Predominance Of A*02011 and identification of HLA-A*0231. *Hum. Immunol.* **2000**, *61*, 334–340.
187. Cao, K.; Hollenbach, J.; Shi, X.; Shi, W.; Chopek, M.; Fernandez-Vina, M.A. Analysis of the frequencies of HLA-A, B, and C alleles and haplotypes in the five major ethnic groups of the United States reveals high levels of diversity in these loci and contrasting distribution patterns in these populations. *Hum. Immunol.* **2001**, *62*, 1009–1030.
188. Malm, M.; Vesikari, T.; Blazevic, V. Identification of a First Human Norovirus CD8(+) T Cell Epitope Restricted to HLA-A(*)0201 Allele. *Front. Immunol.* **2018**, *9*, 2782.
189. Kirby, A.; Iturriza-Gomara, M. Norovirus diagnostics: Options, applications and interpretations. *Expert Rev. Anti-Infect. Ther.* **2012**, *10*, 423–433.
190. Kirby, A.; Gurgel, R.Q.; Dove, W.; Vieira, S.C.; Cunliffe, N.A.; Cuevas, L.E. An evaluation of the RIDASCREEN and IDEIA enzyme immunoassays and the RIDAQUICK immunochromatographic test for the detection of norovirus in faecal specimens. *J. Clin. Virol.* **2010**, *49*, 254–257.
191. Ambert-Balay, K.; Pothier, P. Evaluation of 4 immunochromatographic tests for rapid detection of norovirus in faecal samples. *J. Clin. Virol.* **2013**, *56*, 194–198.
192. Bruggink, L.D.; Dunbar, N.L.; Marshall, J.A. Evaluation of the updated RIDA(R)QUICK (Version N1402) immunochromatographic assay for the detection of norovirus in clinical specimens. *J. Virol. Methods* **2015**, *223*, 82–87.
193. de Bruin, E.; Duizer, E.; Vennema, H.; Koopmans, M.P. Diagnosis of Norovirus outbreaks by commercial ELISA or RT-PCR. *J. Virol. Methods* **2006**, *137*, 259–264.
194. Beersma, M.F.; Sukhrie, F.H.; Bogerman, J.; Verhoef, L.; Mde Melo, M.; Vonk, A.G.; Koopmans, M. Unrecognized norovirus infections in health care institutions and their clinical impact. *J. Clin. Microbiol.* **2012**, *50*, 3040–3045.
195. Sakamaki, N.; Ohiro, Y.; Ito, M.; Makinodan, M.; Ohta, T.; Suzuki, W.; Takayasu, S.; Tsuge, H. Bioluminescent enzyme immunoassay for the detection of norovirus capsid antigen. *Clin. Vaccine Immunol.* **2012**, *19*, 1949–1954.
196. Yasuura, M.; Fujimaki, M. Detection of Extremely Low Concentrations of Biological Substances Using Near-Field Illumination. *Sci. Rep.* **2016**, *6*, 39241.
197. Gairard-Dory, A.C.; Degot, T.; Hirschi, S.; Schuller, A.; Leclercq, A.; Renaud-Picard, B.; Gourieux, B.; Kessler, R. Clinical usefulness of oral immunoglobulins in lung transplant recipients with norovirus gastroenteritis: A case series. *Transplant. Proc.* **2014**, *46*, 3603–3605.
198. Dai, Y.C.; Wang, Y.Y.; Zhang, X.F.; Tan, M.; Xia, M.; Wu, X.B.; Jiang, X.; Nie, J. Evaluation of anti-norovirus IgY from egg yolk of chickens immunized with norovirus P particles. *J. Virol. Methods* **2012**, *186*, 126–131.

199. Dai, Y.C.; Zhang, X.F.; Tan, M.; Huang, P.; Lei, W.; Fang, H.; Zhong, W.; Jiang, X. A dual chicken IgY against rotavirus and norovirus. *Antivir. Res.* **2013**, *97*, 293–300.
200. Vega, C.G.; Bok, M.; Vlasova, A.N.; Chattha, K.S.; Fernandez, F.M.; Wigdorovitz, A.; Parreno, V.G.; Saif, L.J. IgY antibodies protect against human Rotavirus induced diarrhea in the neonatal gnotobiotic piglet disease model. *PLoS ONE* **2012**, *7*, e42788.
201. Ehrlich, P.H.; Moustafa, Z.A.; Harfeldt, K.E.; Isaacson, C.; Ostberg, L. Potential of primate monoclonal antibodies to substitute for human antibodies: Nucleotide sequence of chimpanzee Fab fragments. *Hum. Antib. Hybrid.* **1990**, *1*, 23–26.
202. Ogata, N.; Ostberg, L.; Ehrlich, P.H.; Wong, D.C.; Miller, R.H.; Purcell, R.H. Markedly prolonged incubation period of hepatitis B in a chimpanzee passively immunized with a human monoclonal antibody to the a determinant of hepatitis B surface antigen. *Proc. Natl. Acad. Sci. USA* **1993**, *90*, 3014–3018.
203. Coppieters, K.; Dreier, T.; Silence, K.; de Haard, H.; Lauwereys, M.; Casteels, P.; Beirnaert, E.; Jonckheere, H.; Van de Wiele, C.; Staelens, L.; et al. Formatted anti-tumor necrosis factor alpha VHH proteins derived from camelids show superior potency and targeting to inflamed joints in a murine model of collagen-induced arthritis. *Arthritis Rheum.* **2006**, *54*, 1856–1866.
204. Vincke, C.; Loris, R.; Saerens, D.; Martinez-Rodriguez, S.; Muyldermans, S.; Conrath, K. General strategy to humanize a camelid single-domain antibody and identification of a universal humanized nanobody scaffold. *J. Biol. Chem.* **2009**, *284*, 3273–3284.
205. Holliger, P.; Hudson, P.J. Engineered antibody fragments and the rise of single domains. *Nat. Biotechnol.* **2005**, *23*, 1126–1136.
206. Pant, N.; Hultberg, A.; Zhao, Y.; Svensson, L.; Pan-Hammarstrom, Q.; Johansen, K.; Pouwels, P.H.; Ruggeri, F.M.; Hermans, P.; Frenken, L.; et al. Lactobacilli expressing variable domain of llama heavy-chain antibody fragments (lactobodies) confer protection against rotavirus-induced diarrhea. *J. Infect. Dis.* **2006**, *194*, 1580–1588.
207. van der Vaart, J.M.; Pant, N.; Wolvers, D.; Bezemer, S.; Hermans, P.W.; Bellamy, K.; Sarker, S.A.; van der Logt, C.P.; Svensson, L.; Verrips, C.T.; et al. Reduction in morbidity of rotavirus induced diarrhoea in mice by yeast produced monovalent llama-derived antibody fragments. *Vaccine* **2006**, *24*, 4130–4137.
208. Tokuhara, D.; Alvarez, B.; Mejima, M.; Hiroiwa, T.; Takahashi, Y.; Kurokawa, S.; Kuroda, M.; Oyama, M.; Kozuka-Hata, H.; Nochi, T.; et al. Rice-based oral antibody fragment prophylaxis and therapy against rotavirus infection. *J. Clin. Investig.* **2013**, *123*, 3829–3838.
209. Vega, C.G.; Bok, M.; Vlasova, A.N.; Chattha, K.S.; Gomez-Sebastian, S.; Nunez, C.; Alvarado, C.; Lasa, R.; Escribano, J.M.; Garaicoechea, L.L.; et al. Recombinant monovalent llama-derived antibody fragments (VHH) to rotavirus VP6 protect neonatal gnotobiotic piglets against human rotavirus-induced diarrhea. *PLoS Pathog.* **2013**, *9*, e1003334.
210. Chagla, Z.; Quirt, J.; Woodward, K.; Neary, J.; Rutherford, C. Chronic norovirus infection in a transplant patient successfully treated with enterally administered immune globulin. *J. Clin. Virol.* **2013**, *58*, 306–308.
211. Ronchetti, A.M.; Henry, B.; Ambert-Balay, K.; Pothier, P.; Decroocq, J.; Leblond, V.; Roos-Weil, D. Norovirus-related chronic diarrhea in a patient treated with alemtuzumab for chronic lymphocytic leukemia. *BMC Infect. Dis.* **2014**, *14*, 239.
212. Nilsson, M.; Hedlund, K.O.; Thorhagen, M.; Larson, G.; Johansen, K.; Ekspong, A.; Svensson, L. Evolution of human calicivirus RNA in vivo: Accumulation of mutations in the protruding P2 domain of the capsid leads to structural changes and possibly a new phenotype. *J. Virol.* **2003**, *77*, 13117–13124.
213. Koromyslova, A.; Tripathi, S.; Morozov, V.; Schroten, H.; Hansman, G.S. Human norovirus inhibition by a human milk oligosaccharide. *Virology* **2017**, *508*, 81–89.
214. Weichert, S.; Koromyslova, A.; Singh, B.K.; Hansman, S.; Jennewein, S.; Schroten, H.; Hansman, G.S. Structural Basis for Norovirus Inhibition by Human Milk Oligosaccharides. *J. Virol.* **2016**, *90*, 4843–4848.
215. Schroten, H.; Hanisch, F.G.; Hansman, G.S. Human Norovirus Interactions with Histo-Blood Group Antigens and Human Milk Oligosaccharides. *J. Virol.* **2016**, *90*, 5855–5859.
216. Dolin, R.; Blacklow, N.R.; DuPont, H.; Formal, S.; Buscho, R.F.; Kasel, J.A.; Chames, R.P.; Hornick, R.; Chanock, R.M. Transmission of acute infectious nonbacterial gastroenteritis to volunteers by oral administration of stool filtrates. *J. Infect. Dis.* **1971**, *123*, 307–312.
217. Parrino, T.A.; Schreiber, D.S.; Trier, J.S.; Kapikian, A.Z.; Blacklow, N.R. Clinical immunity in acute gastroenteritis caused by Norwalk agent. *N. Engl. J. Med.* **1977**, *297*, 86–89.
218. Wyatt, R.G.; Dolin, R.; Blacklow, N.R.; DuPont, H.L.; Buscho, R.F.; Thornhill, T.S.; Kapikian, A.Z.; Chanock, R.M. Comparison of three agents of acute infectious nonbacterial gastroenteritis by cross-challenge in volunteers. *J. Infect. Dis.* **1974**, *129*, 709–714.
219. Cannon, J.L.; Lindesmith, L.C.; Donaldson, E.F.; Saxe, L.; Baric, R.S.; Vinje, J. Herd immunity to GII.4 noroviruses is supported by outbreak patient sera. *J. Virol.* **2009**, *83*, 5363–5374.
220. Simmons, K.; Gambhir, M.; Leon, J.; Lopman, B. Duration of immunity to norovirus gastroenteritis. *Emerg. Infect. Dis.* **2013**, *19*, 1260–1267.
221. Hogle, J.M. Poliovirus cell entry: Common structural themes in viral cell entry pathways. *Annu. Rev. Microbiol.* **2002**, *56*, 677–702.
222. Adeyemi, O.O.; Nicol, C.; Stonehouse, N.J.; Rowlands, D.J. Increasing Type 1 Poliovirus Capsid Stability by Thermal Selection. *J. Virol.* **2017**, *91*, e01586-16.
223. Singharoy, A.; Polavarapu, A.; Joshi, H.; Baik, M.H.; Ortoleva, P. Epitope fluctuations in the human papillomavirus are under dynamic allosteric control: A computational evaluation of a new vaccine design strategy. *J. Am. Chem. Soc.* **2013**, *135*, 18458–18468.
224. LoBue, A.D.; Thompson, J.M.; Lindesmith, L.; Johnston, R.E.; Baric, R.S. Alphavirus-adjuvanted norovirus-like particle vaccines: Heterologous, humoral, and mucosal immune responses protect against murine norovirus challenge. *J. Virol.* **2009**, *83*, 3212–3227.

225. Parra, G.I.; Bok, K.; Taylor, R.; Haynes, J.R.; Sosnovtsev, S.V.; Richardson, C.; Green, K.Y. Immunogenicity and specificity of norovirus Consensus GII.4 virus-like particles in monovalent and bivalent vaccine formulations. *Vaccine* **2012**, *30*, 3580–3586.
226. Ramani, S.; Atmar, R.L.; Estes, M.K. Epidemiology of human noroviruses and updates on vaccine development. *Curr. Opin. Gastroenterol.* **2014**, *30*, 25–33.
227. Ramani, S.; Neill, F.H.; Ferreira, J.; Treanor, J.J.; Frey, S.E.; Topham, D.J.; Goodwin, R.R.; Borkowski, A.; Baehner, F.; Mendelman, P.M.; et al. B-Cell Responses to Intramuscular Administration of a Bivalent Virus-Like Particle Human Norovirus Vaccine. *Clin. Vaccine Immunol.* **2017**, *24*, e00571-16.
228. Ball, J.M.; Graham, D.Y.; Opekun, A.R.; Gilger, M.A.; Guerrero, R.A.; Estes, M.K. Recombinant Norwalk virus-like particles given orally to volunteers: Phase I study. *Gastroenterology* **1999**, *117*, 40–48.
229. El-Kamary, S.S.; Pasetti, M.F.; Mendelman, P.M.; Frey, S.E.; Bernstein, D.I.; Treanor, J.J.; Ferreira, J.; Chen, W.H.; Sublett, R.; Richardson, C.; et al. Adjuvanted intranasal Norwalk virus-like particle vaccine elicits antibodies and antibody-secreting cells that express homing receptors for mucosal and peripheral lymphoid tissues. *J. Infect. Dis.* **2010**, *202*, 1649–1658.
230. Treanor, J.J.; Atmar, R.L.; Frey, S.E.; Gormley, R.; Chen, W.H.; Ferreira, J.; Goodwin, R.; Borkowski, A.; Clemens, R.; Mendelman, P.M. A novel intramuscular bivalent norovirus virus-like particle vaccine candidate--reactogenicity, safety, and immunogenicity in a phase 1 trial in healthy adults. *J. Infect. Dis.* **2014**, *210*, 1763–1771.
231. Periwal, S.B.; Kourie, K.R.; Ramachandaran, N.; Blakeney, S.J.; DeBruin, S.; Zhu, D.; Zamb, T.J.; Smith, L.; Udem, S.; Eldridge, J.H.; et al. A modified cholera holotoxin CT-E29H enhances systemic and mucosal immune responses to recombinant Norwalk virus-virus like particle vaccine. *Vaccine* **2003**, *21*, 376–385.
232. Parra, G.I.; Green, K.Y. Sequential Gastroenteritis Episodes Caused by 2 Norovirus Genotypes. *Emerg. Infect. Dis.* **2014**, *20*, 1016–1018.
233. Tamminen, K.; Huhti, L.; Vesikari, T.; Blazevic, V. Pre-existing immunity to norovirus GII-4 virus-like particles does not impair de novo immune responses to norovirus GII-12 genotype. *Viral Immunol.* **2013**, *26*, 167–170.
234. Kobayashi, M.; Ohfuji, S.; Fukushima, W.; Maeda, A.; Maeda, K.; Fujioka, M.; Hirota, Y. Immunogenicity and reactogenicity of a monovalent inactivated 2009 influenza A vaccine in adolescents: With special reference to pre-existing antibody. *J. Pediatr.* **2012**, *160*, 632–637.e1.
235. Knuchel, M.C.; Marty, R.R.; Morin, T.N.; Ilter, O.; Zuniga, A.; Naim, H.Y. Relevance of a pre-existing measles immunity prior immunization with a recombinant measles virus vector. *Hum. Vaccines Immunother.* **2013**, *9*, 599–606.
236. Saif, M.A.; Bonney, D.K.; Bigger, B.; Forsythe, L.; Williams, N.; Page, J.; Babiker, Z.O.; Guiver, M.; Turner, A.J.; Hughes, S.; et al. Chronic norovirus infection in pediatric hematopoietic stem cell transplant recipients: A cause of prolonged intestinal failure requiring intensive nutritional support. *Pediatr. Transplant.* **2011**, *15*, 505–509.
237. Wingfield, T.; Gallimore, C.I.; Xerry, J.; Gray, J.J.; Klapper, P.; Guiver, M.; Blanchard, T.J. Chronic norovirus infection in an HIV-positive patient with persistent diarrhoea: A novel cause. *J. Clin. Virol.* **2010**, *49*, 219–222.
238. Nicollier-Jamot, B.; Pico, V.; Pothier, P.; Kohli, E. Molecular cloning, expression, self-assembly, antigenicity, and seroepidemiology of a genogroup II norovirus isolated in France. *J. Clin. Microbiol.* **2003**, *41*, 3901–3904.
239. Herrmann, J.E.; Blacklow, N.R.; Matsui, S.M.; Lewis, T.L.; Estes, M.K.; Ball, J.M.; Brinker, J.P. Monoclonal antibodies for detection of Norwalk virus antigen in stools. *J. Clin. Microbiol.* **1995**, *33*, 2511–2513.
240. Hardy, M.E.; Tanaka, T.N.; Kitamoto, N.; White, L.J.; Ball, J.M.; Jiang, X.; Estes, M.K. Antigenic mapping of the recombinant Norwalk virus capsid protein using monoclonal antibodies. *Virology* **1996**, *217*, 252–261.
241. Hale, A.D.; Tanaka, T.N.; Kitamoto, N.; Ciarlet, M.; Jiang, X.; Takeda, N.; Brown, D.W.; Estes, M.K. Identification of an epitope common to genogroup 1 "norwalk-like viruses". *J. Clin. Microbiol.* **2000**, *38*, 1656–1660.
242. Kitamoto, N.; Tanaka, T.; Natori, K.; Takeda, N.; Nakata, S.; Jiang, X.; Estes, M.K. Cross-reactivity among several recombinant calicivirus virus-like particles (VLPs) with monoclonal antibodies obtained from mice immunized orally with one type of VLP. *J. Clin. Microbiol.* **2002**, *40*, 2459–2465.
243. Jiang, X.; Matson, D.O.; Velazquez, F.R.; Calva, J.J.; Zhong, W.M.; Hu, J.; Ruiz-Palacios, G.M.; Pickering, L.K. Study of Norwalk-related viruses in Mexican children. *J. Med. Virol.* **1995**, *47*, 309–316.
244. Yoda, T.; Terano, Y.; Suzuki, Y.; Yamazaki, K.; Oishi, I.; Kuzuguchi, T.; Kawamoto, H.; Utagawa, E.; Takino, K.; Oda, H.; et al. Characterization of Norwalk virus GI specific monoclonal antibodies generated against Escherichia coli expressed capsid protein and the reactivity of two broadly reactive monoclonal antibodies generated against GII capsid towards GI recombinant fragments. *BMC Microbiol.* **2001**, *1*, 24.
245. Higo-Moriguchi, K.; Shirato, H.; Someya, Y.; Kurosawa, Y.; Takeda, N.; Taniguchi, K. Isolation of cross-reactive human monoclonal antibodies that prevent binding of human noroviruses to histo-blood group antigens. *J. Med. Virol.* **2014**, *86*, 558–567.
246. Atmar, R.L.; Opekun, A.R.; Gilger, M.A.; Estes, M.K.; Crawford, S.E.; Neill, F.H.; Ramani, S.; Hill, H.; Ferreira, J.; Graham, D.Y. Determination of the 50% human infectious dose for Norwalk virus. *J. Infect. Dis.* **2014**, *209*, 1016–1022.

247. Hurwitz, A.M.; Huang, W.; Kou, B.; Estes, M.K.; Atmar, R.L.; Palzkill, T. Identification and Characterization of Single-Chain Antibodies that Specifically Bind GI Noroviruses. *PLoS ONE* **2017**, *12*, e0170162.
248. Treanor, J.; Dolin, R.; Madore, H.P. Production of a monoclonal antibody against the Snow Mountain agent of gastroenteritis by in vitro immunization of murine spleen cells. *Proc. Natl. Acad. Sci. USA* **1988**, *85*, 3613–3617.
249. Yoda, T.; Terano, Y.; Suzuki, Y.; Yamazaki, K.; Oishi, I.; Utagawa, E.; Shimada, A.; Matsuura, S.; Nakajima, M.; Shibata, T. Characterization of monoclonal antibodies generated against Norwalk virus GII capsid protein expressed in Escherichia coli. *Microbiol. Immunol.* **2000**, *44*, 905–914.
250. Almanza, H.; Cubillos, C.; Angulo, I.; Mateos, F.; Caston, J.R.; van der Poel, W.H.; Vinje, J.; Barcena, J.; Mena, I. Self-assembly of the recombinant capsid protein of a swine norovirus into virus-like particles and evaluation of monoclonal antibodies cross-reactive with a human strain from genogroup II. *J. Clin. Microbiol.* **2008**, *46*, 3971–3979.
251. Li, X.; Zhou, R.; Wang, Y.; Sheng, H.; Tian, X.; Li, H.; Qiu, H. Identification and characterization of a native epitope common to norovirus strains GII/4, GII/7 and GII/8. *Virus Res.* **2009**, *140*, 188–193.
252. Hansman, G.S.; Taylor, D.W.; McLellan, J.S.; Smith, T.J.; Georgiev, I.; Tame, J.R.; Park, S.Y.; Yamazaki, M.; Gondaira, F.; Miki, M.; et al. Structural basis for broad detection of genogroup II noroviruses by a monoclonal antibody that binds to a site occluded in the viral particle. *J. Virol.* **2012**, *86*, 3635–3646.
253. Crawford, S.E.; Ajami, N.; Parker, T.D.; Kitamoto, N.; Natori, K.; Takeda, N.; Tanaka, T.; Kou, B.; Atmar, R.L.; Estes, M.K. Mapping broadly reactive norovirus genogroup I and II monoclonal antibodies. *Clin. Vaccine Immunol.* **2015**, *22*, 168–177.
254. Dong, X.; Moyer, M.M.; Yang, F.; Sun, Y.P.; Yang, L. Carbon Dots' Antiviral Functions Against Noroviruses. *Sci. Rep.* **2017**, *7*, 519.
255. Rogers, J.D.; Ajami, N.J.; Fryszczyn, B.G.; Estes, M.K.; Atmar, R.L.; Palzkill, T. Identification and characterization of a peptide affinity reagent for detection of noroviruses in clinical samples. *J. Clin. Microbiol.* **2013**, *51*, 1803–1808.
256. Yoda, T.; Suzuki, Y.; Terano, Y.; Yamazaki, K.; Sakon, N.; Kuzuguchi, T.; Oda, H.; Tsukamoto, T. Precise characterization of norovirus (Norwalk-like virus)-specific monoclonal antibodies with broad reactivity. *J. Clin. Microbiol.* **2003**, *41*, 2367–2371.
257. Moore, M.D.; Bobay, B.G.; Mertens, B.; Jaykus, L.A. Human Norovirus Aptamer Exhibits High Degree of Target Conformation-Dependent Binding Similar to That of Receptors and Discriminates Particle Functionality. *mSphere* **2016**, *1*, e00298-16.
258. Shiota, T.; Okame, M.; Takanashi, S.; Khamrin, P.; Takagi, M.; Satou, K.; Masuoka, Y.; Yagyu, F.; Shimizu, Y.; Kohno, H.; et al. Characterization of a broadly reactive monoclonal antibody against norovirus genogroups I and II: Recognition of a novel conformational epitope. *J. Virol.* **2007**, *81*, 12298–12306.
259. Parra, G.I.; Azure, J.; Fischer, R.; Bok, K.; Sandoval-Jaime, C.; Sosnovtsev, S.V.; Sander, P.; Green, K.Y. Identification of a broadly cross-reactive epitope in the inner shell of the norovirus capsid. *PLoS ONE* **2013**, *8*, e67592.
260. Zheng, L.; Wang, W.; Liu, J.; Chen, X.; Li, S.; Wang, Q.; Huo, Y.; Qin, C.; Shen, S.; Wang, M. Characterization of a Norovirus-specific monoclonal antibody that exhibits wide spectrum binding activities. *J. Med. Virol.* **2018**, *90*, 671–676.
261. Katpally, U.; Wobus, C.E.; Dryden, K.; Virgin, H.W.t.; Smith, T.J. Structure of antibody-neutralized murine norovirus and unexpected differences from viruslike particles. *J. Virol.* **2008**, *82*, 2079–2088.
262. Malm, M.; Tamminen, K.; Heinimaki, S.; Vesikari, T.; Blazevic, V. Functionality and avidity of norovirus-specific antibodies and T cells induced by GII.4 virus-like particles alone or co-administered with different genotypes. *Vaccine* **2018**, *36*, 484–490.
